# Conformational Transduction Amplification in a Biomimetic Polyelectrolyte for Ultrasensitive Imaging of Iron Metabolism

**DOI:** 10.1002/advs.76185

**Published:** 2026-06-19

**Authors:** Yeqiang Zhou, Danqi Yang, Yiwei Wang, Shuangyan Li, Fan Fan, Jiayu Zou, Cheng Zhang, Yang Liu, Hong Tan, Mingming Ding

**Affiliations:** ^1^ College of Polymer Science and Engineering National Key Laboratory of Advanced Polymer Materials Sichuan University Chengdu China

**Keywords:** allosteric polyelectrolytes, conformational transition, near‐infrared fluorescence, nonlinear fluorescence response, ultrasensitive bioimaging

## Abstract

Proteins exert sophisticated functions through stimulus‐responsive conformational changes. Mimicking this structural control and functional integration in synthetic, water‐soluble polymers has been a fundamental challenge, largely due to the conflict between charge repulsion and conformational order. Here, we report a peptidomimetic polyelectrolyte that overcomes this limitation, maintaining stable and well‐defined helical and sheet‐like conformations across the entire physiological pH range despite its high charge density. This intrinsic conformational order creates through‐space conjugated carbonyl clusters that function as non‐classical chromophores, enabling excitation‐dependent near‐infrared fluorescence with an exceptionally large Stokes shift. More importantly, the sheet‐like conformation enables cooperative, multidentate chelation of Fe^3^^+^ ions, which triggers an allosteric transition to a helix and results in nonlinear and ultrasensitive fluorescence quenching. Leveraging this unique “conformational transduction amplification” mechanism, we achieve real‐time visualization and tracking of iron ion distribution and metabolic pathways at subcellular and whole‐organism levels. This work establishes a paradigm of allosteric control in synthetic polyelectrolytes, opening avenues for the design of intelligent biomimetic materials for advanced sensing and imaging.

## Graphical abstract

This work designs a peptide‐free polyelectrolyte with stable, switchable conformations and near‐infrared fluorescence in water. It harnesses a cooperative Fe^3^^+^‐triggered sheet‐to‐helix transition to generate nonlinear fluorescence quenching, enabling the ultrasensitive, real‐time visualization of Fe^3^^+^ ion distribution and metabolic trafficking at subcellular levels in living cells and mapping its systemic pathway in vivo.

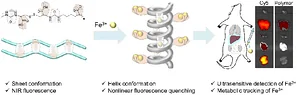

## 1 Introduction

Proteins epitomize nature's exquisite integration of structure and function, wherein dynamic and responsive molecular conformations serve as primary determinants of biological activity [1, 2]. These conformational transitions govern critical biological processes such as molecular transport, signaling, and catalysis, as exemplified by the allosteric regulation of hemoglobin and calcium‐induced structural switching of calmodulin [3, 4, 5, 6, 7]. Fluorescent proteins further demonstrate how subtle conformational shifts, often modulated by protonation or metal coordination, directly modulate optical output [8, 9, 10]. These phenomena have long inspired efforts to engineer synthetic polymers capable of stimulus‐triggered conformational changes, thereby enabling the rational design of intelligent materials for sensing, diagnostics, and therapeutics.

Realizing this in aqueous media, however, remains a fundamental challenge, particularly for charged systems. While natural polypeptides stabilize ordered conformations (e.g., α‐helices and β‐sheets) through a delicate balance of backbone hydrogen bonding and side‐chain interactions, synthetic polyelectrolytes, including classical poly(amino acid)s such as polylysine or polyglutamate, typically adopt disordered states in aqueous solution due to overwhelming electrostatic repulsion [11]. Although strategies such as charge shielding [12], side‐chain spacing [13], or metal coordination have been devised to promote ordering in peptide‐based polymers [14], these approaches often require complex synthesis or compromise chemical functionality. Critically, to our knowledge, no general design exists for a water‐soluble, non‐peptide polyelectrolyte that retains both a stable, well‐defined secondary structure across physiological pH and the ability to undergo conformational switching in response to a specific biological stimulus.

Here, we report a class of peptidomimetic polyelectrolytes, poly(ureido acid)s (PUAs), that directly challenge this prevailing design constraint. Despite the complete absence of peptide bonds and a high density of ionizable carboxyl groups, PUAs adopt stable sheet‐like and helical conformations in water across a wide pH range (1–12). This intrinsic conformational stability in a highly water‐soluble charged polymer is unprecedented. Notably, these ordered conformations give rise to strong, conformation‐sensitive, nonclassical fluorescence that is tunable into the near‐infrared (NIR) region, with emission maxima reaching 830 nm, among the longest wavelengths reported for non‐conjugated polymer. Moreover, the sheet‐like conformation of PUA enables a cooperative iron‐coordination involving both backbone urea and pendant carboxyl groups. This binding triggers a sheet‐to‐helix conformational switch, a transduction mechanism that results in a highly nonlinear, ultrasensitive fluorescence quenching. Leveraging this response, we demonstrate real‐time visualization and tracking of iron ion metabolism at subcellular and sub‐tissue levels. This work not only advances fundamental understanding of structure‐function relationships of biological macromolecules but also establishes a new generation of functional, fluorescent aliphatic polymers with tremendous potential for biomedical applications.

## 2 Results and Discussion

### 2.1 Synthesis of PUA With pH‐Adjustable Ordered Conformations in Water

To access water‐soluble, conformationally ordered polyelectrolytes without relying on complex polypeptide synthesis, we developed a straightforward and scalable route to anionic PUAs. Starting from protected lysine and cystine derivatives, we employed a room‐temperature interfacial polymerization, followed by mild deprotection, to obtain the target polymers in high yield (Figure S1). Proton nuclear magnetic resonance (^1^H NMR) spectra confirmed the presence of characteristic lysine methylene protons (δ 1.86, 1.52, and 2.13 ppm) and cystine‐derived methylene protons adjacent to disulfide bonds (δ 3.04–3.24 ppm) (Figure S2). Fourier‐transform infrared (FTIR) spectroscopy revealed a strong carbonyl stretching band between 1600–1700 cm^−1^ (urea C═O stretch) and a broad N─H stretch between 3700–3100 cm^−1^, confirming the urea‐based backbone. The presence of the pendant carboxylic acid functionality was evidenced by a distinct C═O stretching band at 1700–1760 cm^−1^ (Figure S3). Gel permeation chromatography (GPC) showed a monomodal molecular weight distribution with a polydispersity index (Đ) of 1.2–1.4 (Figure S4). The number‐average molecular weights were further corroborated by end‐group analysis via ^1^H NMR (Figures S5 and S6 and Table S1).

**FIGURE 1:**
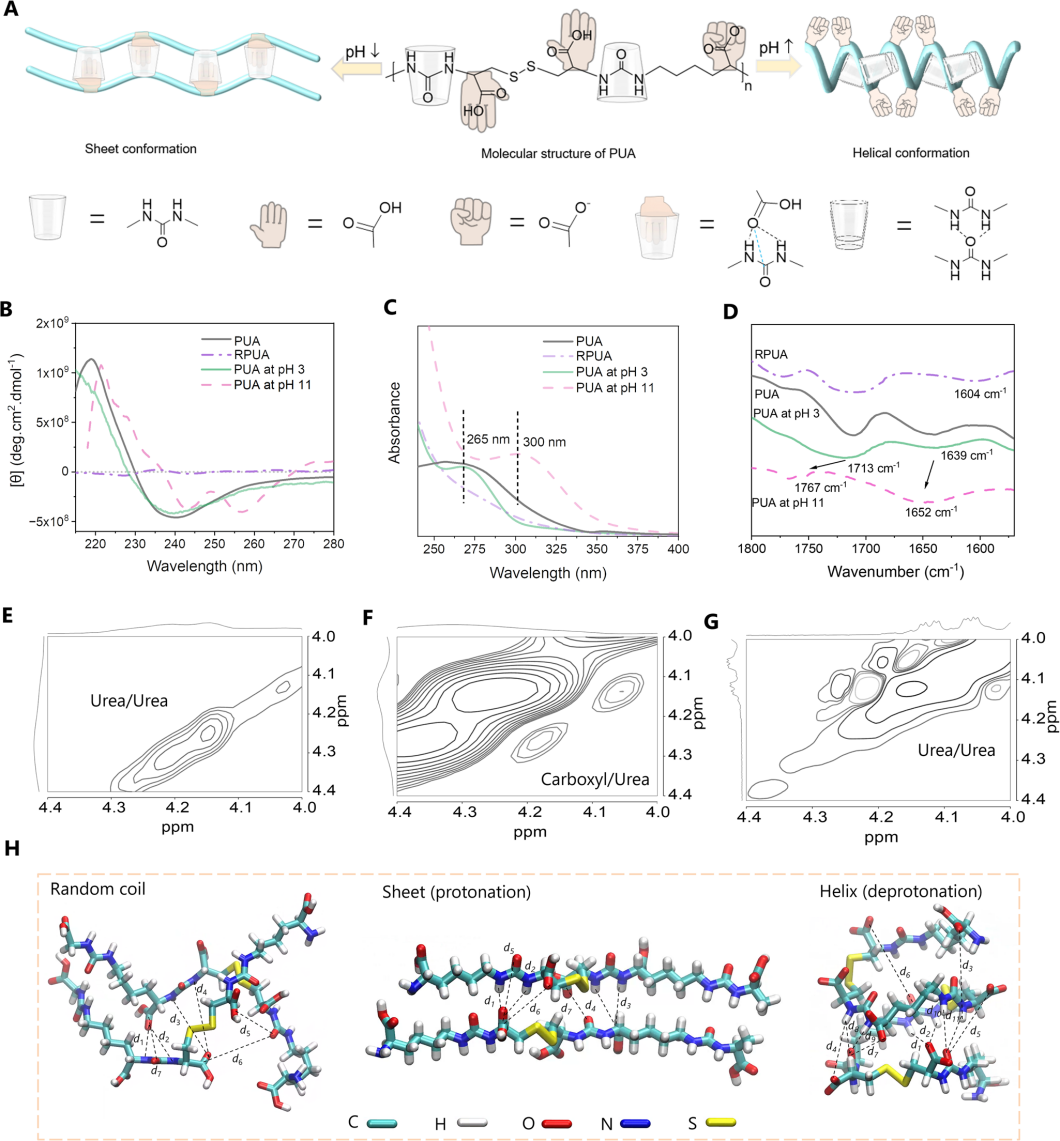
PUA with pH‐adjustable ordered conformations in water. (A) Schematic illustration of the molecular design and pH‐adjustable conformations. (B) CD spectra of PUA and RPUA aqueous solutions at various pH values. (C) UV–vis absorbance spectra of PUA and RPUA aqueous solutions at various pH values. (D) FTIR spectra of PUA and RPUA aqueous solutions at various pH values. (E–G) Partial 1H‐1H NOESY spectra containing major NOEs of RPUA (E) and PUA (F) in D_2_O, and PUA under alkaline conditions (G). (H) Conformations of PUA and RPUA obtained from molecular simulations.

The designed PUA maintained well‐defined secondary structures in water despite its high charge density (Figure 1A), in contrast to classical polyanions like polyglutamate, which adopt ordered conformations only upon side‐chain neutralization [15, 16]. Circular dichroism (CD) spectroscopy revealed that, under acidic and neutral conditions, PUA exhibited a spectral signature characteristic of a β‐sheet‐like conformation [17], with a positive peak near 220 nm and a single negative peak at ∼240 nm (Figure 1B). Remarkably, under alkaline conditions, the CD spectrum transformed into a double‐minimum pattern with negative peaks at 243 and 256 nm, indicative of a helical conformation [18, 19, 20]. In contrast, the racemic control polymer (RPUA) showed only a weak, featureless CD signal across all pH values, consistent with a random coil state. These results demonstrate that PUA not only formed stable ordered conformations but also underwent pH‐induced order‐to‐order transitions between sheet and helical states.

To elucidate the mechanism underlying this unprecedented conformational stability and switching, we performed variable‐pH ultraviolet‐visible (UV–vis) spectroscopy. PUA exhibited a strong absorption band centered at 265 nm, attributed to *n*–*π* ^*^ transitions of carbonyl groups [21]. The band intensified and red‐shifted to 300 nm upon alkalinization (Figure 1C). This shift is consistent with the altered electronic environment of carbonyl groups, likely arising from deprotonation of the carboxyl side chains [22]. FTIR spectroscopy provided direct evidence for changes in these interactions. In the sheet state (pH *3*), the urea C═O stretching vibration appeared at 1639 cm^−1^, a pronounced redshift from its typical position (∼1690–1700 cm^−^^1^ for free urea) [23]. Concurrently, the C═O stretch of the protonated carboxyl group was observed at 1713 cm^−1^ (Figure 1D), also significantly red‐shifted relative to a non‐hydrogen‐bonded carboxylic acid [24, 25, 26]. These concerted shifts confirm strong interactions between the backbone urea and carboxyl side chains [23, 27], which act as the primary driving force stabilizing the sheet‐like conformation. In the helical state (pH *11*), the carboxylate C═O stretch shifted to 1767 cm^−^^1^, consistent with deprotonation and increased electron density [25], while the urea C═O band shifted to 1652 cm^−^^1^ (Figure 1D). This shift indicates disruption of the urea‐carboxyl interaction and strengthening of urea‐urea hydrogen bonding along the polymer backbone [23], facilitating the transition to a helical structure stabilized by internal backbone hydrogen bonds. This mechanism is reminiscent of a peptide α‐helix, albeit within a non‐peptide framework.

To gain further insight into the intramolecular forces that stabilized the distinct conformations of PUA, we performed ^1^H‐^1^H nuclear Overhauser effect spectroscopy (NOESY). For the racemic control polymer RPUA, the NOESY spectrum showed strong cross‐peaks only between the urea amide protons (H8, H9) (Figure 1E and Figure S7). In contrast, the spectra of PUA at pH *3* (sheet‐like structure) revealed strong correlations between the lysine‐derived spacer (H4, H5, and H6) and the urea backbone protons (Figure 1F and Figure S7). This pattern signified a sheet‐like architecture where the aliphatic side chains are in close spatial proximity to the backbone, providing direct evidence for the urea‐carboxyl interaction proposed from the FTIR data. Upon switching to pH *11* (helical state), the NOE correlations characteristic of the sheet‐like structure largely disappeared (Figure 1G and Figure S7). Instead, a new set of strong cross‐peaks emerged, primarily between urea N─H protons (H8, H9) and the protons near the urea groups (H2, H3). These findings confirm the disruption of the urea‐carboxyl interaction and the enhanced interactions among backbone urea groups under alkaline conditions, leading to the formation of the helical structure.

To better understand the pH‐dependent conformational transition, we conducted density functional theory (DFT) simulations (Figure 1H). When the carboxyl groups were protonated, the PUA chain adopted a sheet‐like structure. The urea N─H protons remained in close proximity to the carbonyl oxygen of the side‐chain carboxyl groups, with an average N─H···O═C distance of 3.13 Å. Upon deprotonation, electrostatic repulsion between the negatively charged side chains disrupted the urea‐carboxyl contacts, increasing their average distance to 8.75 Å. Concurrently, the backbone contracted, bringing adjacent urea groups into closer proximity, with an average C═O···H─N distance of 3.09 Å (Table S2). Unlike classical polyelectrolytes such as polyglutamate or polyaspartate, which adopt a random coil conformation under side‐chain charge repulsion [28], the same repulsive force within PUA selectively breaks the urea‐carboxyl interactions while simultaneously strengthening intrachain urea‐urea interactions, thereby facilitating the formation of a rigid helical structure.

### 2.2 Ordered Conformations Enable Aqueous NIR Luminescence

**FIGURE 2:**
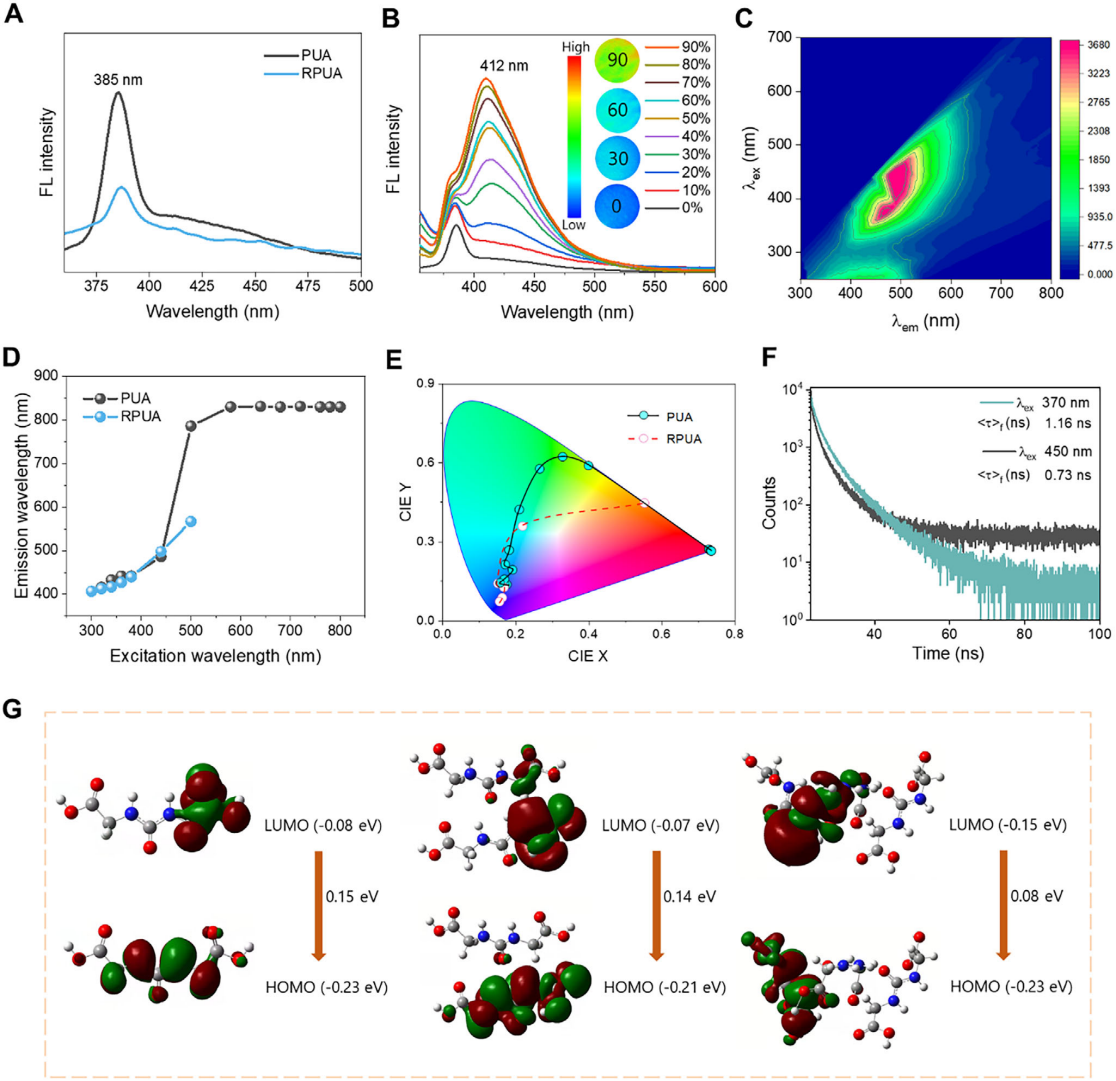
Conformation‐dependent NIR clusteroluminescence. (A) Emission spectra of PUA dilute aqueous solutions at λ_ex_ 340 nm. (B) Fluorescence spectra of PUA in H_2_O/THF mixtures with 0%–90% THF fractions. The PUA emission images in mixes with varying THF percentages (0%, 30%, 60%, and 90%) are displayed in the insets. (C) 3D fluorescence spectrum image of a PUA film. (D) The relation between λ_ex_ and λ_em_ for PUA and RPUA. (E) CIE coordinates diagram of PUA and RPUA at different λ_ex_. (F) Lifetime decay profiles of PUA (*λ*_ex_ = 370, 450 nm). (G) Frontier molecular orbitals of optimized excited‐state geometries of PUA. HOMO and LUMO were calculated for monomer, dimer, and trimer of PUA in the excited states. The blue and red isosurfaces represent the orbital wavefunctions with opposite signs.

Harnessing non‐classical luminescence in biomedical aqueous environments remains a challenge, as emission from most systems is quenched by hydrogen bonding with water [29, 30]. The ordered conformations of PUA in water present a unique solution to this problem. We propose that the tight chain packing enforced by the sheet and helical structures brings the urea or carboxyl carbonyl groups into close proximity, facilitating the formation of clustered chromophores [31]. Spectroscopic analysis confirmed this hypothesis. In dilute aqueous solution at pH *3*, PUA exhibited a strong, broad fluorescence emission with a maximum at 385 nm when excited at 340 nm (Figure 2A). The fluorescence intensity of PUA was more than twice that of the racemic RPUA control under identical conditions, highlighting the critical role of ordered conformations in promoting fluorescence emission. Increasing the polymer concentration or adding a poor solvent (e.g., tetrahydrofuran) to the aqueous solution led to a significant fluorescence enhancement and the emergence of a new, red‐shifted emission band at 412 nm (Figure 2B and Figure S8). This concentration‐ and aggregation‐dependent spectral shift is characteristic of the clustering‐triggered emission (CTE) mechanism [32].

The ordered conformation of PUA not only activated aqueous fluorescence but also enabled remarkably red‐shifted emission through controlled molecular packing. Three‐dimensional fluorescence spectroscopy of concentrated solutions and solid‐state films revealed that PUA exhibited a far stronger and broader emission profile than the disordered RPUA control, with a distinct emission band extending into the NIR region and peaking at 830 nm (Figure 2C–E and Figures S9 and S10). This represents the longest emission wavelength reported to date for a non‐conjugated polymer [33]. This long‐wavelength emission displayed clear excitation‐dependent emission (EDE) behavior, indicating the presence of multiple, distinct emissive species [29]. Time‐resolved fluorescence measurements revealed different lifetimes under various excitation wavelengths (Figure 2F), confirming the existence of such species [30]. We attribute these species to varying degrees of through‐space conjugation (TSC) among the tightly packed carbonyl clusters. To substantiate the TSC mechanism, we analyzed the UV–vis absorption and excitation spectra of PUA solutions as a function of polymer concentration. With increasing concentration, the intensity of the UV absorption peak at 266 nm (*n*–*π* ^*^ transition) grew substantially, and a new, lower‐energy band emerged at 353 nm (Figure S11). Correspondingly, the peak at 305 nm in the excitation spectrum was red‐shifted to 342 nm, accompanied by a marked enhancement of the band at 362 nm (Figure S12). These observations suggest that aggregation brought carbonyl groups from adjacent chains into close spatial proximity, strengthening *n*–*π* ^*^ interactions and enhancing electron delocalization, thereby narrowing the optical gap and facilitating long‐wavelength emission [33].

Furthermore, time‐dependent DFT calculations revealed that as the polymer chains aggregated, the average distance between carbonyl groups decreased from 4.88 to 4.07 Å (Figure S13), and the energy gap between the highest occupied and lowest unoccupied molecular orbitals (HOMO‐LUMO) was substantially reduced (Figure 2G). These findings verified that aggregation‐induced TSC is the fundamental mechanism enabling NIR luminescence. The combination of ultralong‐wavelength emission and an exceptionally large Stokes shift (>110 nm) distinguished PUA from conventional fluorescent materials (Figure S9). This Stokes shift substantially exceeds that of standard NIR dyes (e.g., cyanine dyes and indocyanine green, typically < 30 nm) [34]. A large Stokes shift minimizes self‐absorption and cross‐talk between excitation and emission channels, which is critical for achieving superior tissue penetration depth and a high signal‐to‐noise ratio in complex biological environments [35, 36].

### 2.3 Conformation‐Dependent Nonlinear Fluorescence Quenching via Allosteric Switching

**FIGURE 3:**
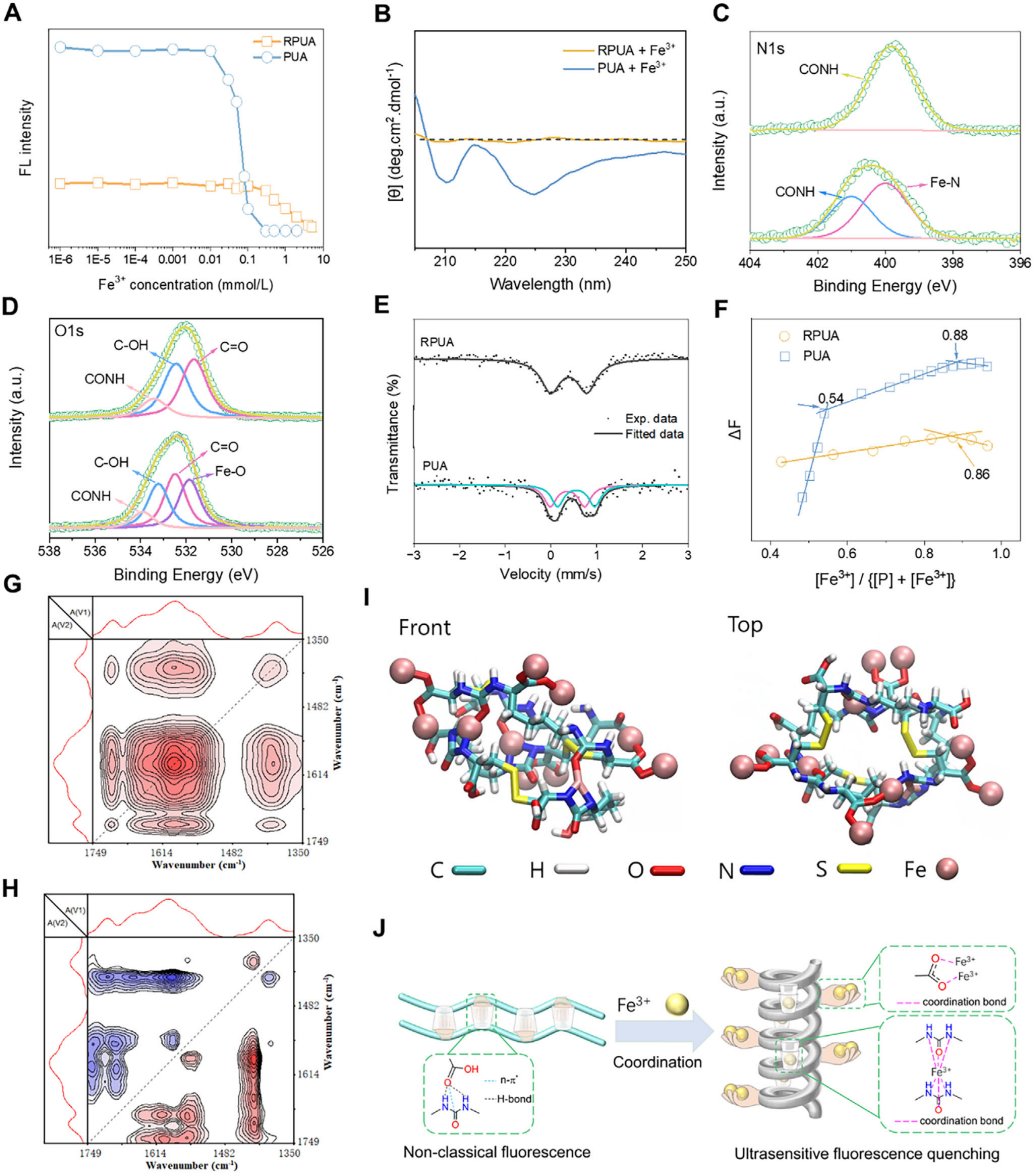
Conformation‐dependent nonlinear fluorescence quenching via allosteric switching. (A) Dependence of fluorescence intensity on Fe^3+^ concentration in PUA and RPUA aqueous solutions. (B) CD spectra of PUA and RPUA coordinated with Fe^3+^. (C,D) High‐resolution XPS spectra of N 1s (C) and O 1s (D) for PUA and PUA‐Fe^3+^. (E) Mössbauer spectra of PUA‐Fe^3+^ and RPUA‐Fe^3+^ at 300 K. (F) Job's plot of PUA and RPUA with Fe^3+^. [P] + [Fe^3+^] = 5 × 10^−7^ m, where “[P]” represents the repeating unit, λ_ex_ = 340 nm. (G, H) Synchronous (G) and asynchronous (H) 2D correlation maps generated from the 1749‐1350 cm^−1^ region of FTIR spectra of PUA‐Fe^3+^. Blue denotes negative correlation, and red denotes a positive correlation; a higher color intensity signifies a stronger correlation. (I) Optimized binding geometry of the complex at the B3LYP/6‐311+G (2d, p) level of theory. (J) Schematic illustration of the Fe^3+^ ion‐induced conformational transition.

In proteins, the binding of metal ions can induce changes in conformation and in the chemical or electronic environment of the chromophore, resulting in variations in luminescence performance [37]. Given the high affinity of Fe^3^^+^ (with its vacant orbitals and high charge‐to‐radius ratio) for the urea and carboxyl groups [38], we investigated its interaction with PUA. PUA exhibited exceptional selectivity for Fe^3^^+^ over a panel of biologically relevant metal ions, causing near‐complete fluorescence quenching accompanied by a significant reduction in fluorescence lifetime (Figures S14 and S15), consistent with a dynamic quenching process [39]. Crucially, the quenching response was profoundly dependent on the polymer's initial conformation. The disordered RPUA control exhibited a weak, linear decrease in fluorescence intensity with increasing Fe^3^^+^ concentration, similar to other reported nonclassical fluorescent materials [38]. In stark contrast, PUA demonstrated a sharp, sigmoidal quenching curve, with over 80% of the fluorescence extinguished within an exceptionally narrow concentration window (50–90 µmol/L) (Figure 3A). This highly nonlinear, cooperative response signifies an ultrasensitive detection capability unprecedented in non‐conjugated polymer probes.

We hypothesized that the distinct quenching behaviors of PUA and RPUA correlated with their different conformational states. To test this hypothesis, we analyzed the polymer‐iron complexes using CD spectroscopy. For PUA, Fe^3^^+^ titration induced a shift of the CD pattern into double minima at 210 and 224 nm, characteristic of a helical conformation [40, 41, 42, 43, 44]. This confirms that Fe^3^^+^ binding triggered a sheet‐to‐helix conformational transition. In contrast, RPUA‐Fe^3+^ complex retained a random coil structure (Figure 3B). Isothermal titration calorimetry (ITC) quantified the dramatic thermodynamic differences. Although both interactions were exothermic and enthalpy‐driven [45], PUA exhibited a significantly higher binding affinity for Fe^3^^+^, with a dissociation constant (K_D_) of 0.532 µm, nearly an order of magnitude lower than that of RPUA (K_D_ = 3.43 µm) (Figure S16). Furthermore, the binding stoichiometries of PUA and RPUA were 1.58 and 0.54, respectively. This distinct stoichiometry, together with the affinity difference, implies a cooperative, multidentate binding mode in the conformationally ordered PUA, contrasting with that in the disordered RPUA [46]. X‐ray photoelectron spectroscopy (XPS) of the PUA‐Fe^3^^+^ complex showed distinct peaks corresponding to both Fe─O (531.8 eV) and Fe─N (400.0 eV) bonds, whereas only Fe─O bonds (533.9 eV) were detected in the RPUA complex (Figure 3C,D and Figures S17 and S18). This provides direct evidence that in PUA, Fe^3^^+^ was chelated by both carboxyl oxygen and ureylene oxygen/nitrogen atoms, in contrast to RPUA, where coordination was limited to oxygen atoms. Mössbauer spectroscopy further corroborated the existence of two distinct iron coordination environments in the PUA complex (asymmetric doublet), vs. a single, symmetric environment in the RPUA complex (Figure 3E) [47]. The stepwise nature of this synergistic chelation was resolved through Job's plot analysis of the fluorescence titration (Figure 3F). For PUA, quenching occurred in two distinct phases: a sharp phase (inflection at 0.5) followed by a gradual saturation phase. The results indicate an initial, high‐affinity binding stoichiometric ratio of 2:1 between urea groups and Fe^3^^+^, followed by lower‐affinity binding to carboxylates. In contrast, RPUA exhibited only a single phase of slow quenching, consistent with binding to carboxyl groups alone.

To elucidate the sequence and mode of Fe^3^^+^ coordination, we employed coordination‐dependent two‐dimensional infrared (2D IR) correlation spectroscopy. For PUA, the synchronous 2D IR map exhibited strong auto‐peaks and positive cross‐peaks correlating the urea C═O stretch (∼1605 cm^−^^1^), the carboxyl C═O stretch (∼1715 cm^−^^1^), and the COO^−^ symmetric stretch (∼1410 cm^−^^1^) (Figure 3G). This indicates that these vibrational modes changed concurrently upon Fe^3^^+^ addition. Crucially, the asynchronous map revealed a more detailed sequence, negative cross‐peaks at coordinates such as (1585, 1410) and (1715, 1605) cm^−1^ demonstrate that changes in the urea carbonyl vibrations preceded those of the carboxyl groups (Figure 3H). This provides direct spectroscopic evidence that Fe^3^^+^ binds first to the pre‐organized urea sites in the sheet structure, initiating the coordination event. In contrast, the 2D IR spectra for RPUA showed correlation features only for carboxyl vibrations (e.g., at (1715, 1410) cm^−^^1^), with no detectable involvement of urea bands (Figure S19), suggesting that Fe^3^^+^ interacted solely with the carboxyl groups. Complementary FTIR spectroscopy provided quantitative insights into the coordination geometry (Figure S20). Upon Fe^3^^+^ binding, PUA exhibited significant redshifts in the N─H (∼18 cm^−^^1^) and urea C═O (∼35 cm^−^^1^) stretching bands, and a new characteristic band emerged at 1651 cm^−^^1^, signifying urea carbonyl coordination and the formation of the helical conformation [48]. No such changes occurred in RPUA. Furthermore, the separation between the asymmetric and symmetric COO^−^ stretches, Δν = ν_as_(COO^−^)—ν_s_(COO^−^), was in the range of 148–177 cm^−^^1^ for both complexes (Figure S20). This value suggests a bidentate, bridging coordination mode where a single carboxyl group bridged two Fe^3^^+^ ions, consistent with the 2:1 stoichiometry inferred from Job's plot analysis (Figure 3F).

To investigate the mechanisms behind coordination‐driven conformational changes, we performed molecular simulations. Energy‐optimized structure and free energy landscape analysis confirmed that the PUA‐Fe^3+^ complex exhibited a helical conformation, where each Fe^3^^+^ ion was chelated by two urea carbonyl groups, forming a periodic urea‐Fe‐urea framework that stabilized the helix (Figure 3I). Concurrently, the carboxyl side chains extended outward, participating in bridging coordination with Fe^3^^+^, consistent with our spectroscopic data. These results demonstrate that Fe^3^^+^ engaged in a cooperative, multidentate chelation in PUA, which triggered the sheet‐to‐helix transition (Figure 3J). This is the principal cause of the nonlinear, ultrasensitive quenching of PUA fluorescence. The ability of the ordered sheet‐like architecture to provide specific, high‐affinity chelation pockets unavailable to the disordered state provides a design principle for allosteric signal amplification.

### 2.4 Intracellular Fe^3^^+^ Imaging With Conformation‐Activated Ultrasensitivity

Capitalizing on its conformation‐dependent, cooperative response, we developed PUA into a ratiometric fluorescent probe for real‐time tracking of Fe^3^^+^ in living cells. To correct for variations in probe concentration and instrumental factors, we conjugated the polymer with a cyanine dye (Cy5) whose fluorescence is independent of Fe^3^^+^, creating a dual‐emission system. The fluorescence intensity ratio (*I*_polymer_/*I*_Cy5_) provided an internal reference for Fe^3^^+^ detection (Figure S21). The RPUA‐based probe exhibited a linear and gradual decrease in the intensity ratio across a broad Fe^3^^+^ concentration range, whereas the PUA probe exhibited a sharp, sigmoidal quenching response, with the signal effectively extinguished within a narrow window in the low micromolar range. This translates to a limit of detection for Fe^3^^+^ that is 3 to 10 times lower than those of widely reported fluorescent probes [49, 50, 51].

**FIGURE 4:**
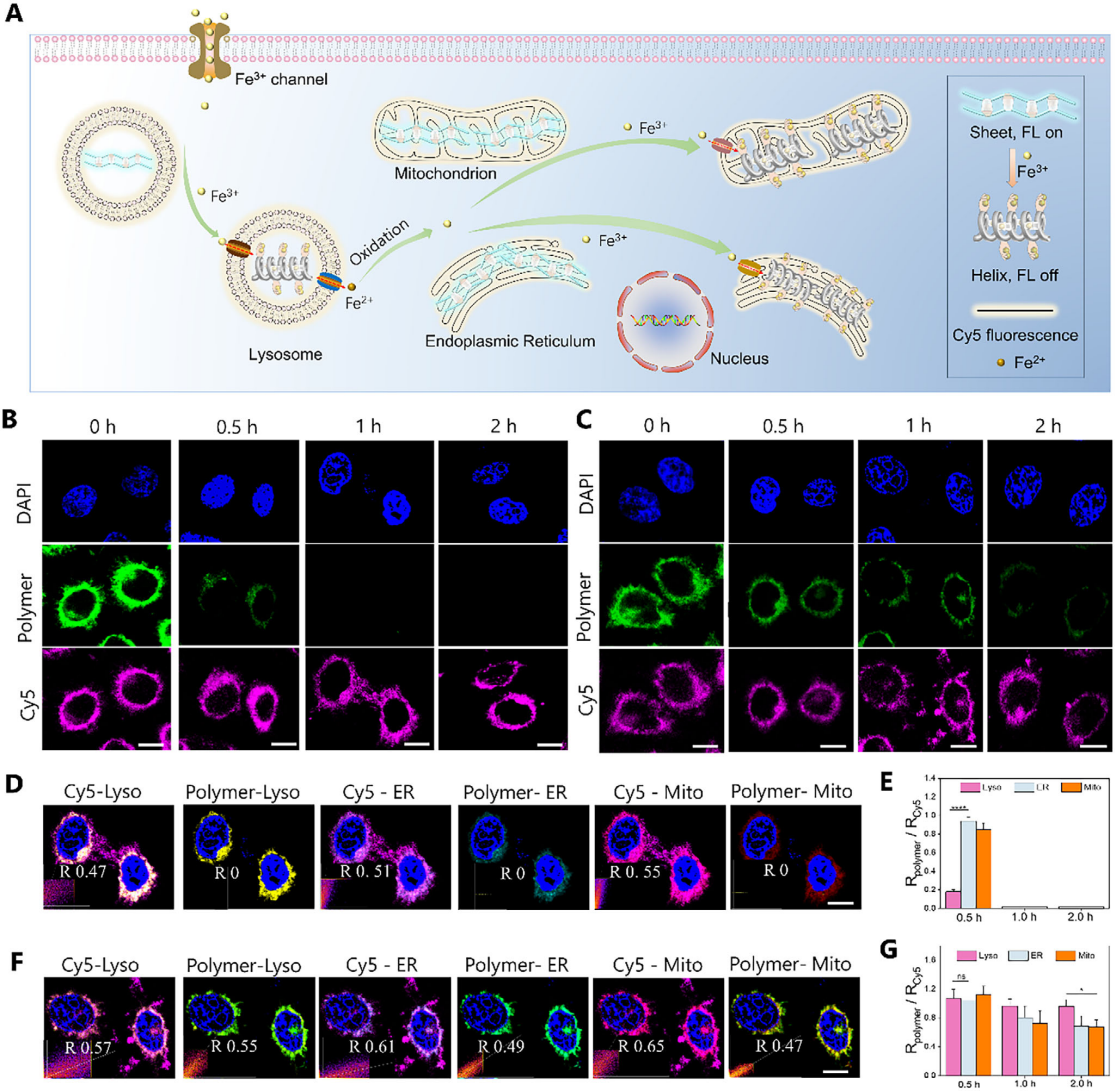
Real‐time sensing of iron ion distribution in different organelles. (A) Schematic illustration of Cy5‐PUA monitoring the metabolism of Fe^3+^ ions in different subcellular regions. (B,C) CLSM images of MCF‐7 cells pretreated with Cy5‐PUA (B) and Cy5‐RPUA (C) (1 mg/mL) after 0.5, 1, and 2 h of incubation with FAC. Scale bars 20 µm. (D) CLSM images and Pearson's correlation coefficients showing the co‐localization of the PUA with different organelles in MCF‐7 cells after 1 h of incubation with PUA. Scale bars 20 µm. (E) Temporal profiles of the mean fluorescence intensity ratio R_polymer_/R_Cy5_ of the corresponding subcellular imaging shown in (D) by statistical analysis. Data are presented as mean ± standard deviation (*n*  =  3 independent samples). Statistical significance was calculated by the two‐tailed paired Student's t‐test, ns: no significant; ^*^: *p* < 0.05; ^**^: *p* < 0.01; ^***^: *p* < 0.001; ^****^: p < 0.0001. (F) CLSM images and Pearson's correlation coefficients showing the co‐localization of the RPUA with different organelles in MCF‐7 cells after 1 h of incubation with PUA. Scale bars 20 µm. (G) Temporal profiles of the mean fluorescence intensity ratio R_polymer_/R_Cy5_ of the corresponding subcellular imaging shown in (F) by statistical analysis. Data are presented as mean ± standard deviation (*n*  =  3 independent samples). Statistical significance was calculated by one‐way ANOVA with Tukey's multiple comparisons test, ns: no significant; ^*^: *p* < 0.05; ^**^: *p* < 0.01; ^***^: *p* < 0.001; ^****^: *p* < 0.0001.

We next evaluated the performance of the probes in living MCF‐7 breast cancer cells. After a 1‐h incubation, both the PUA and control RPUA showed bright intracellular fluorescence from both channels, confirming cellular uptake and an initial “ON” state (Figure 4A–C). Subsequent treatment with a low concentration of exogenous Fe^3^^+^ (10 µm) elevated intracellular total iron levels to 0.23∼0.42 µmol/gprot, as quantified by a standard colorimetric assay (Figure S22). Confocal laser scanning microscopy (CLSM) revealed a rapid and pronounced quenching of the PUA channel signal, indicating a switching to the “OFF” state, while the RPUA control showed a markedly attenuated response. The fluorescence of Cy5 reference channel remained invariant in both cases, suggesting that the observed quenching originated specifically from iron binding to the polymer. To confirm this binding, we compared cell viability upon challenge with ferric ammonium citrate (FAC) in the presence and absence of the polymer. The CCK‐8 assay demonstrated a significant rescue of cell viability following polymer treatment (Figure S23). These findings confirm that the polyelectrolyte sequestered intracellular labile iron pools, which enabled not only an ultrasensitive “ON‐OFF” sensing of intracellular Fe^3^^+^ but also mitigation of iron‐induced cytotoxicity.

To identify the subcellular distribution of iron ions, we performed a co‐localization analysis of Cy5 and polymer fluorescence with lysosome tracker (LT), mitochondria tracker (MT) and endoplasmic reticulum tracker (ERT). Pearson's correlation coefficients (PCCs) were utilized to assess the distribution of iron ions across different organelles at various time points. Notably, after a 30 min incubation, LT showed a co‐localization of 0.66 with Cy5, while the co‐localization with PUA was significantly lower, at only 0.12. In contrast, MT and ERT exhibited similar colocalization ratios with both Cy5 and PUA (Figure S24), suggesting an initial accumulation of iron ions in lysosomes. At 60 min, the PCCs of Cy5 with the three organelles were consistently around 0.5, whereas PUA exhibited negligible co‐localization with any organelles, indicating that the iron ions had equilibrated across all three organelles, rendering the polymer in an “off” state (Figure 4D,E). Control experiments performed at lower probe concentrations yielded consistent results, thereby excluding potential polymer concentration effects on iron ion tracking (Figures S25–S27). These observations align with known pathways of iron metabolism, where lysosomes are the first organelles to receive extracellular iron ions, and the iron ions released from lysosomes are subsequently taken up by the endoplasmic reticulum and mitochondria [52, 53, 54]. In the case of RPUA, the PCCs with the three organelles remained relatively constant for both the polymer and Cy5, implying that the iron concentration did not reach levels sufficient to completely quench RPUA fluorescence (Figure 4F,G and Figure S24). These findings highlight the potential of PUA as an ultrasensitive probe for the detection and imaging of trace amounts of iron ions at the subcellular level in real time.

### 2.5 Real‐Time Tracking of Systemic Iron Metabolism In Vivo

**FIGURE 5:**
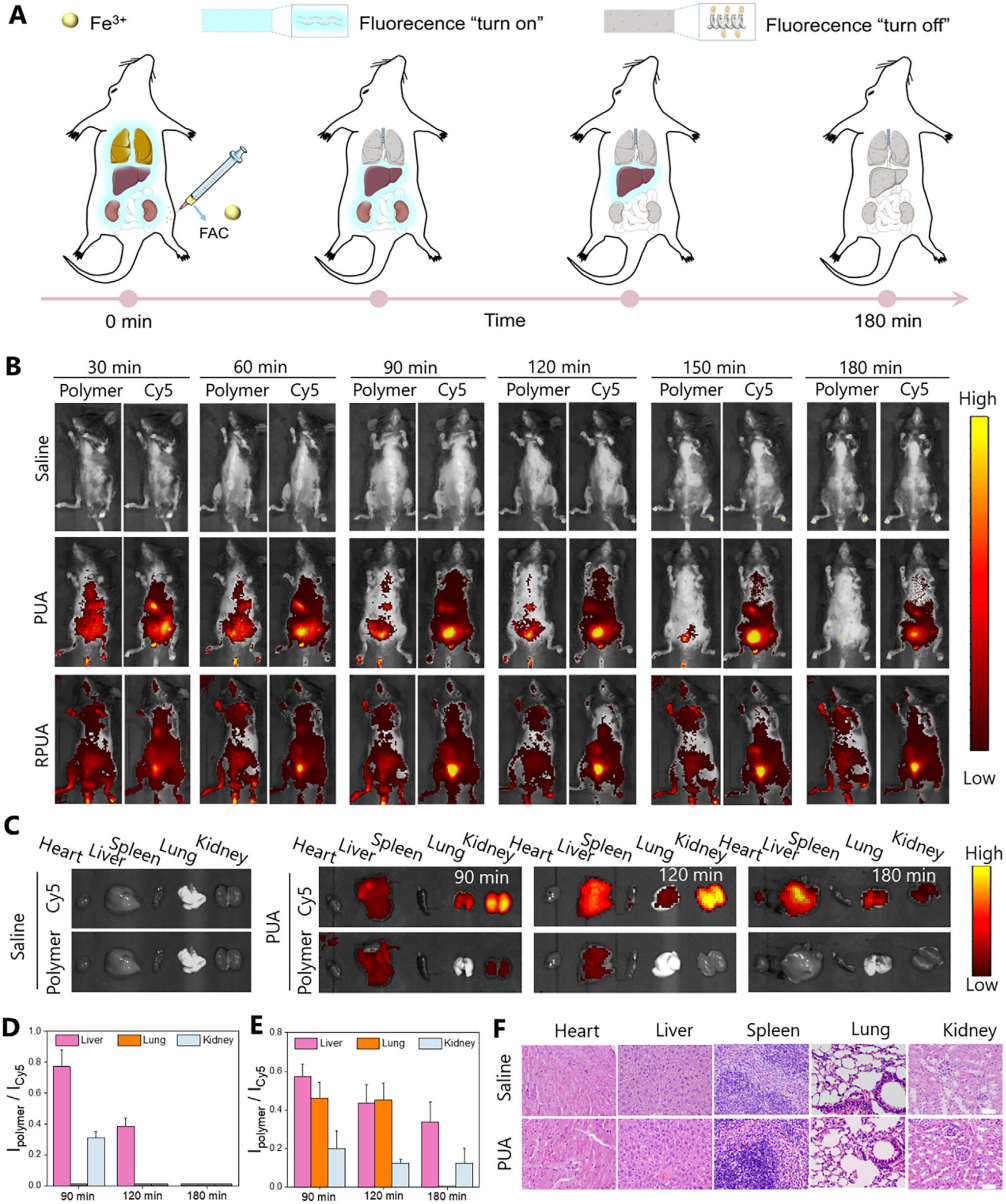
Real‐time tracking of systemic iron metabolism in vivo. (A) Schematic illustration of the PUA monitoring the metabolic pathway of Fe^3+^ ions in vivo. (B) In vivo fluorescence images at different time points after intraperitoneal injection of FAC following pretreatment with PUA and RPUA. Mice treated with saline were set as controls, λ_ex_ = 480 nm, λ_em_ = 520 nm. PUA, λ_ex_ = 520 nm, λ_em_ = 790 nm; RPUA, λ_ex_ = 480 nm, λ_em_ = 520 nm. (C) Ex vivo fluorescence images of major organs from mice in different groups at the indicated time points. Temporal profiles of the mean fluorescence intensity ratio *I*_polymer_/*I*_Cy5_ for PUA (D) and RPUA (E) detected in the liver, lung, and kidney of the corresponding ex vivo organ imaging shown (Figure 5C and Figure S30) by statistical analysis. Data are presented as mean ± standard deviation (*n*  =  5 independent mice). (F) Representative hematoxylin and eosin (H&E)‐stained images of tissues from major organs. Scale bars 50 µm.

The dynamic distribution and metabolism of metal ions are central to physiology and disease, yet real‐time, non‐invasive tracking with high spatiotemporal resolution remains a formidable challenge [37, 55]. Many existing probes are limited by rapid clearance, poor tissue penetration, or insufficient sensitivity for tracking low‐concentration fluxes in vivo [56, 57]. PUA addresses these limitations through its combination of NIR, a large Stokes shift, exceptional water solubility, and, critically, its conformation‐driven ultrasensitivity. To evaluate its potential for in vivo application, we assessed the photostability of the polymer under physiological conditions. Notably, prolonged light‐exposure time and continuous photobleaching under physiological conditions caused little change in the fluorescence intensity (Figure S28). This photostability makes PUA a promising tool for in vivo bioimaging and sensing. Next, we performed real‐time in vivo imaging in mice injected intraperitoneally with Cy5‐conjugated PUA (Figure 5A). Following administration of FAC, the PUA signal exhibited a progressive, organ‐specific quenching sequence: fluorescence in the lungs largely disappeared within 90 min, followed by the kidneys (∼120 min), and finally the liver (∼180 min) (Figure 5B–E and Figures S29–S31). To validate this iron distribution, we measured iron content in the organs at different times and performed Perls' Prussian blue staining analysis of tissue sections (Figures S32 and S33). The results confirmed iron deposition in the lungs, kidneys, and liver at 90, 120, and 180 min, respectively. This spatiotemporal profile implies that the metabolic process of iron ions in vivo may initiate in the pulmonary circulation and proceed through renal and hepatic processing [58, 59, 60]. In contrast, the control RPUA probe, with its linear, low‐sensitivity response, showed only weak and diffuse signal attenuation, failing to resolve this sequential metabolic route. These findings demonstrate that the nonlinear, cooperative quenching mechanism of PUA can convert subtle ionic changes into optical signals that enable real‐time spatiotemporal mapping of systemic iron trafficking. In addition, histopathological analysis of major organs harvested after the imaging studies revealed no signs of acute inflammation or tissue damage (Figure 5F). In a separate long‐term toxicity study, mice receiving multi‐dose administration of PUA exhibited no significant body weight loss, changes in serum biochemical markers, or histological abnormalities, confirming the biocompatibility of PUA for in vivo applications (Figure S34). Collectively, these results establish PUA as a safe, highly sensitive macromolecular platform for in vivo bioimaging and theranostics of iron metabolism‐related diseases.

## 3 Conclusion

In summary, we have designed and synthesized a class of protein‐mimetic polyelectrolytes. By encoding stable, pH‐switchable secondary structures within a non‐peptide backbone, we resolved the long‐standing conflict among charge repulsion, conformational order, and fluorescence properties in aqueous media. This polymer exhibited unique photophysical properties, including conformation‐sensitive, non‐classical fluorescence that extended into the NIR region with an exceptionally large Stokes shift. Crucially, we discovered that the sheet‐like conformation of the polymer enabled a synergistic, multidentate coordination with Fe^3^^+^ involving both backbone urea and pendant carboxyl groups. This specific binding triggered a sheet‐to‐helix transition and resulted in a nonlinear fluorescence quenching response. Compared with conventional fluorescent probes, this “conformational transduction amplification” mechanism enhanced sensitivity by an order‐of‐magnitude. Exploiting this ultrasensitive response, we demonstrated real‐time visualization of iron trafficking at subcellular levels and successfully tracked the metabolic pathways of iron ions in vivo. This work provides new insight into macromolecule‐metal interactions and offers an intelligent polymeric material for advanced biosensing and medical imaging.

## 4 Experimental Section

The supporting information included a full description of the materials and methods used.

## Author Contributions

**Yeqiang Zhou**: data curation, investigation, conceptualization, writing – original draft. **Mingming Ding**: writing – review and editing, conceptualization, funding acquisition, project administration. **Yang Liu**: validation. **Fan Fan**: formal analysis. **Jiayu Zou**: visualization. **Danqi Yang**: formal analysis, data curation, writing – original draft. **Cheng Zhang**: methodology. **Hong Tan**: supervision. **Yiwei Wang**: formal analysis. **Shuangyan Li**: visualization.

## Ethics Statement

All animal studies were approved by the Institutional Animal Care and Use Committee of Sichuan University (Approval No. KS2023301) and were conducted in strict accordance with established ethical guidelines for animal research.

## Conflicts of Interest

The authors declare no conflicts of interest.

## Supporting information




**Supporting File**: advs76185‐sup‐0001‐SuppMat.pdf.

## Data Availability

The main text and the supplemental materials contain all of the data produced during this investigation. The corresponding author can provide materials and reagents upon request.
