# Supplementary material for: Conformational Transduction Amplification in a Biomimetic Polyelectrolyte for Ultrasensitive Imaging of Iron Metabolism
Source: Adv Sci (Weinh). 2026 Jun 19;13(51):e76185. doi: 10.1002/advs.76185 (PMC13336357; doi:10.1002/advs.76185)
Supplement: Supplementary file 1 [file ADVS-13-e76185-s001.pdf]

Supporting Information

Conformational Transduction Amplification in a Biomimetic Polyelectrolyte for Ultrasensitive Imaging of Iron Metabolism

Yeqiang Zhou[†]*, Danqi Yang*[†]*, Yiwei Wang, Shuangyan Li, Fan Fan, Jiayu Zou, Cheng Zhang, Yang Liu*[*]*, Hong Tan, Mingming Ding*[*]

College of Polymer Science and Engineering, National Key Laboratory of Advanced Polymer Materials, Sichuan University, Chengdu, 610065, China.

E-mail: dmmshx@scu.edu.cn or dmmshx@163.com; liuyang_leon@scu.edu.cn

[†] These authors contributed equally to this work.

Materials and Methods

Materials

Cystine dimethyl ester dihydrochloride (L-Cys·OMe·2HCl, 98%), D-Cystine bis (methyl ester) dihydrochloride (D-Cys·OMe·2HCl) and L-Lysine ethyl ester dihydrochloride (L-Lys·OEt·2HCl) were obtained from Shanghai Hanhong Chemical Technology Co., Ltd. (Shanghai, China). L-Lysine ethyl ester diisocyanate (LDI, 98%) and NaOH (96%) were purchased from Nantong Dahong Chemical Co. Ltd. (Nantong, China). 2-Isocyanatoethyl methacrylate, (IEM, 98%) was purchased from Shanghai Titan Scientlfic Co., Ltd. Stannous octoate was purchased from TEDA Letai Chemical Co. Ltd. (Tianjin, China). *N*,*N*-dimethyl formamide (DMF) and *N*,*N*-dimethylacetamide (DMAC) were obtained from Chengdu Kelong Chemical Co., Ltd. (Chengdu, China). PBS-Tween 20 was obtained from Adamas life. Ferric ammonium citrate (FAC) was obtained from Acros. Dichloromethane (DCM), triethylamine (TEA, AR), and various metal salts (analytical reagent grade, including $FeCl_3·6H_2O$, $CaCl_2$, $CuSO_4·5H_2O$, $AlCl_3·6H_2O$, $MnCl_2·4H_2O$, $Cr(NO_3)_3·9H_2O$, $Cd(NO_3)_2·4H_2O$, $CoCl_2·6H_2O$, $MgSO_4$, NaCl, $FeSO_4·7H_2O$, KCl, LiCl, $AgNO_3$) were attained from Chengdu Changlian Chemical Co., Ltd. Deuterated water (D_2O) was obtained from Sigma-Aldrich (Shanghai, China). Cy5-NHS, Mito-Tracker, LysoTracker, and ER-Tracker were purchased from Beyotime. Unless stated elsewhere, all reagents were obtained from commercial suppliers and used without further purification.

Synthesis of PUA and RPUA

PUA and RPUA were synthesized via interfacial polymerization followed by deprotection. Under an argon atmosphere, L-Cystine dimethyl ester dihydrochloride (L-Cys·OMe·2HCl), D-Cys·OMe·2HCl, or L-Lysine ethyl ester dihydrochloride (L-Lys·OEt·2HCl) was introduced into a round-bottom flask. Subsequently, 60 mL of dichloromethane (DCM) and 250 mL of

phosphate-buffered saline (PBS, pH 8) were added, followed by the addition of 4-8 mL of triethylamine (TEA) with continuous stirring. A solution of LDI in DCM was then slowly added dropwise, allowing the reaction to proceed at room temperature (RT) for 12 h. The resulting solid was dissolved in 1 N NaOH and stirred for additional 12 h. Upon completion of the reaction, the solution was dialyzed against deionized water for 24 h and then lyophilized to obtain PUA and RPUA.

Synthesis of IEM-conjugated polymers

To synthesize IEM-conjugated polymers, polyureido acid ester (PUAE) or racemic polyureido acid ester (RPUAE) (100 mg) was weighed into a round-bottomed flask equipped with a stir bar. Under an inert argon atmosphere, 5 mL of DMF was added to dissolve the polymer. Subsequently, 2.5 mg of IEM was introduced into the reaction mixture via syringe under argon. The reaction mixture was stirred for 24 h at room temperature. Afterward, the mixture was precipitated, washed, filtered, and the product was dried under vacuum for 48 h. The molecular structure of the product was confirmed by ^{1}H NMR spectroscopy (Figure S6). To determine the number of Cys and Lys residues, and the molecular weights of PUA and RPUA, the integral values of specific peaks were analyzed. The integral value of the IEM methyl group (-C$\boldsymbol{H}_3$, 1.87 ppm) was used as a reference. The integral values of the cystine dimethyl ester (-COO-C$\boldsymbol{H}_3$, 3.65 ppm) and the methylene proton of the lysine residues (COO-C$\boldsymbol{H}_2$-CH_3, 4.07 ppm) were obtained and used to calculate the respective quantities (Table S1).

Synthesis of dye-conjugated polymers

The polymers were labeled with the commercially available N-hydroxysuccinimide (NHS) ester-activated fluorophore Cy5 using the following procedure: PUA or RPUA (150 mg) was dissolved in deionized water (5 mL) and mixed with Cy5-NHS (2 mg). The reaction mixture was stirred in the dark for 12 h. Subsequently, the mixture was subjected to dialysis against

phosphate-buffered saline (PBS) to completely remove unbound dye, and then lyophilized to obtain Cy5-labelled polymers.

Structural characterization

Chemical shift assignments were determined using the proton nuclear magnetic resonance (^{1}H NMR). All NMR spectra was recorded on a 400 MHz Bruker Avance III HD spectrometer equipped with a 5 mm TCI CryoProbe at 298 K, using 3 mm NMR tubes. Unless otherwise specified, NMR samples were prepared as 50 mM solutions in 100% D_2O, which had been titrated to the desired pH using aqueous 5 M NaOH. The ^{1}H NMR chemical shifts (δ) were referenced using an internal tetramethylsilane (TMS, δ = 0.0) standard and expressed in parts per million (ppm).

^{1}H-^{1}H nuclear overhauser effect spectroscopy (NOESY) spectrum (240 ms mixing time) was typically acquired using 1024 data points with a sweep width of 4000 Hz in the direct ^{1}H dimension, performed with 4 scans and a relaxation delay of 0.455 ms. Spectra were recorded with a 1 s inter-scan delay and were processed using cosine-squared window functions. Processing and spectral visualization were performed using MestReNova v12.0.0-20080.All the Fourier transform infrared (FTIR) analyses were carried out using a Nicolet 6700 FTIR spectrometer (Thermo Fisher Scientific, USA) under a pure N_2 (100 mL min^{-1}) atmosphere. Background spectra were acquired during the cooling process and subtracted from the corresponding sample spectra. Each spectrum was recorded by performing 32 scans over the wavenumber range of 4,000 to 700 cm^{-1}. Samples were prepared by casting solutions onto KBr plates, followed by drying under vacuum prior to measurement.

Two-dimensional correlation infrared spectroscopy (2D IR) analysis was used to further elucidate the coordination process of Fe^{3+} ion at the molecular level. The 2D IR spectra were processed, calculated, and plotted using 2DCS software. Baseline correction was applied to

ensure the accuracy of results. In the 2D IR spectra, sky-blue and red cross peaks in the contour maps correspond to negative and positive correlation peaks, respectively.

Circular dichroism (CD) spectroscopy

CD measurements were carried out using a J-1500-150 spectrometer (JASCO corporation, Japan). Scans were performed from 280 to 210 nm at RT under continuous nitrogen purge. Aqueous polymer solutions were analyzed using a 1 mm path length quartz cuvette. Each spectrum was averaged, background-corrected, and converted to molar ellipticity $[\theta]$: $[\theta] = (\theta \times 100 \times M_w) / (C \times l)$, where θ represents the observed ellipticity (in degrees) at wavelength λ, M_w is the molecular weight of the amino acid residue, l is the cuvette path length, and C is the sample concentration.

Optical spectroscopy analysis

UV-vis spectra were recorded on a UV2600 spectrophotometer (Techcomp, Ltd., China). Samples were prepared in quartz cuvettes with a 1.00 cm path length, and the wavelength scanning range was 220-700 nm. Photoluminescence excitation and emission spectra, as well as three-dimensional fluorescence signals, were collected using an F-4600 fluorescence spectrometer (Hitachi corporation, Japan), equipped with a Xenon flashlamp as the excitation source.

Quantum mechanics simulations

All molecular calculations were performed using time-dependent density functional theory (TD-DFT) with the B3LYP density functional and the 6-311+G (2d,p) basis set. ABCluster software was utilized to optimize structural conformations using a quantum mechanics (QM) approach. The Multiwfn package was used to calculate electron densities, energy levels, and electronic potential distributions. VMD software was used to visualize the HOMO and LUMO.

Transmission electron microscope (TEM)

The polymer solutions were diluted to a concentration of 0.2 mg mL^{-1}. Samples were prepared by placing a droplet of the diluted polymer solution onto a Formvar-carbon-coated copper grid (200 mesh). After drying, the grid was rinsed with distilled water for several seconds to remove excess liquid, followed by the addition of phosphotungstic acid for negative staining. TEM measurements were carried out using a Zeiss Libra 120 TEM, with an accelerating voltage of 120 kV.

pH measurement

The pH of the solution was measured using a Rex Electric Chemical E-301-QC pH meter. Prior to each test, the pH meter was calibrated with three standard buffer solutions. Following calibration, the electrode was rinsed several times with the pure solvents to eliminate any residual standard solution. Subsequently, the pH of each solution was measured.

Fluorescent lifetime measurement

The samples were placed in a quartz cuvette with a 1.0 cm path length, sealed with a Teflon stopper, and mounted onto the measurement device. The fluorescence time-resolved decay spectra of all constructs were collected at the nanosecond timescale using a FluoroMax-4 Spectrofluorometer (Horiba Jobin Yvon, Kyoto, Japan). The instrument was optically excited with a NanoLED laser and coupled to a time-correlated single-photon counting (TCSPC) acquisition module. For PUA solid, the excitation and emission wavelengths were set at $\lambda_{ex}/\lambda_{em}$ = 370/420 nm and $\lambda_{ex}/\lambda_{em}$ = 450/790 nm.

X-ray photoelectron spectroscopy (XPS)

XPS measurements were carried out on an Axis Supra+ (Kratos Analytical) using Al Kα as the X-ray source. The C 1s, O 1s, N 1s and Fe 2p spectra were collected under an ultra-high vacuum

conditions at 25 °C for all test samples. Survey spectra were acquired with a pass energy of 160 eV and a step size of 1 eV. For high-resolution spectra, a pass energy of 40 eV and a step size of 0.1 eV were used. Peaks centered at 284.8 eV, corresponding to adventitious carbon, were used as a reference for the other binding energies.

Isothermal titration calorimetry (ITC)

ITC experiments were performed using a MicroCal ITC 200 microcalorimeter (GE15 Healthcare). Solutions of PUA or RPUA (500 μM) were titrated into the microcalorimeter cell containing 300 μL of 50 μM $FeCl_3$, via 2 μL injections. The experiments were carried out at 25 °C with a stirring speed of 350 rpm. Binding affinities and thermodynamic parameters were determined using TA Instruments NanoAnalyze Data Analysis software, employing an independent binding site model.

Metal ion selectivity detection

Fluorescence titrations were performed using 1 mg mL^{-1} solution of PUA at room temperature. Concentrated and freshly prepared aliquots of various metal ions (Fe^{3+}, Fe^{2+}, Ca^{2+}, Cu^{2+}, Cu^{+}, Al^{3+}, Zn^{2+}, Mn^{2+}, Cr^{3+}, Cd^{2+}, Co^{2+}, Ni^{2+}, Mg^{2+}, Na^{+}, Sn^{2+}) were added and mixed for 10 min to monitor the change in emission. The fluorescence quenching rate was defined as $\Delta I/I_0$, where ΔI represents the change in fluorescence intensity at 430 nm during titration, and I_0 is the intensity of the maximum fluorescence emission peak at the initial stage.

Mössbauer experiments

The ^{57}Fe Mössbauer spectra were recorded in transmission geometry using a third-generation spectrometer (Ms ~ 96) at 300 K in constant acceleration mode. ^{57}Co/Rh was used as the radiation source. The spectral parameters were quantitatively evaluated using Mosswinn 4.0

software, with least-squares fitting to Lorentzian peaks. Isomer shifts were referenced to α-iron at 300 K.

Fe^{3+} ion titration

In a typical Fe^{3+} titration experiment, the probe solution was titrated with an aqueous stock solution of Fe^{3+} ions in a quartz cell at 25 °C. The fluorescence and absorption spectra were measured 5 min after the addition of the metal ions at each titration point. In the fluorescence titration, the probes were excited at 340 nm and the fluorescence intensity at the maximum emission wavelength (430 nm) was analyzed by nonlinear least-square curve fitting. To determine the stoichiometry of the PUA-Fe^{3+} complex, UV-vis spectra of the Fe^{3+} titration were recorded by adding aliquots of Fe^{3+} solution dropwise into the probe work solution. The absorption intensities at 350 nm were measured and plotted as a function of the molar ratio of $[P_{urea}]/[Fe^{3+}]$, where $[P_{urea}]$ represents the molar concentration of urea in PUA. The mole fraction of $[P_{urea}]/[Fe^{3+}]$ corresponding to the inflection point in the plots was analyzed to determine the binding stoichiometry for each complex.

Job's Plot Measurement

Stock solutions of polymers (0.5 mM) and Fe^{3+} ion (0.5 mM) were prepared in water. A total of 2 mL polymer solutions and Fe^{3+} ion, in the different ratios, were added to quartz cells and thoroughly mixed. The spectra of the resulting solutions were recorded at room temperature. The difference in fluorescence intensity at 385 nm was plotted against the mole fraction of sulfate, at a constant total concentration of 0.5 M in water.

pH titration

To evaluate the pH sensitivity of PUA, pH titrations were carried out by diluting the polymer to a final concentration of 1 μM in various pH buffers, yielding 11 solutions with pH values

ranging from 3.4 to 10.2. Fluorescence emission spectra were recorded with excitation at $\lambda_{ex} = 468$ nm and emission measured at $\lambda_{em} = 385$ and 440 nm. The peak intensity ratio I_{440}/I_{385} was used for the quantitative assessment of pH-sensitive properties. The intensity ratio I_{440}/I_{385} values were plotted as a function of pH and fitted using Origin software. Absorbance scans were obtained over the wavelength range of 250 to 400 nm with a step size of 5 nm.

photostability assay

Polymer samples immersed in Dulbecco's Modified Eagle Medium (DMEM) were continuously excited on an inverted fluorescence microscope (DMi 8, Leica, Germany). Images were acquired every 6 s using an appropriate emission filter and a short exposure time, with the whole system was controlled using LAS X software. The fluorescent intensity of the sample was measured after various durations of continuous irradiation. In addition, the polymer was dissolved in DMEM and continuously illuminated with a 20 W LED. Aliquots (2 mL) were withdrawn at predetermined timepoints, and the fluorescent signal was recorded using a fluorescent spectrophotometer at an excitation wavelength of 340 nm.

Cells and animals

Mouse embryonic fibroblasts (3T3) and human breast cancer cells (MCF-7) were obtained from the American Type Culture Collection (Manassas, VA, USA). These cells were cultured in dulbecco's modified eagle's medium (DMEM) supplemented with 10% fetal bovine serum (FBS) and 1% penicillin-streptomycin (PS). Cultures were maintained in a humidified incubator at 37 °C with a 5% CO_2 atmosphere.

C57BL/6 male SPF mice (6 weeks of age, 20 ± 2 g) were obtained from Beijing Viton Lihua Laboratory Animal Technology Co. Ltd. (Beijing, China). The mice were housed under standard laboratory conditions (constant temperature at 22 °C; 50–60% relative humidity; 12 h light/12 h dark cycle) with free access to water and standard rodent chow. After a period of

acclimation, the mice were randomly assigned to three experimental groups (five mice per group). All procedures were conducted in strict accordance with the guidelines for the care and use of laboratory animals established by the Laboratory Animal Center of Sichuan University.

Confocal laser scanning microscopy (CLSM)

MCF-7 cells were cultured in DMEM medium and seeded into a flat-bottom 6-well plate, allowing for 24 h of adherence. The cells were incubated with culture media containing 0.5 mg/mL or 1 mg/mL Cy5-labeled polymers for different time. Subsequently, the treated cells were washed three times with PBS to remove excess polymers and incubated with 10 μM FAC. After various incubation periods, the cells were washed three times with PBS, fixed with 4% paraformaldehyde for 15 min, and stained with 4′,6-diamidino-2-phenylidole (DAPI) for 10 min. Cell imaging was carried out using CLSM (Olympus FV1000, Nikon A1RMP, Japan) with excitation lasers of 405, 488, and 650 nm for DAPI, polymers and Cy5, respectively.

Co-localization experiments were performed in a dual-channel mode. After various incubation periods, treated cells were washed three times with PBS and stained with MT, LT, ERT for 30 min. The cells were then washed, fixed, and stained as described above. Then, observations were made using a confocal microscope. Pearson's correlation coefficient (PCC) for each group was calculated using ImageJ software.

In vivo and ex vivo imaging study

The hair on the abdomen of mice was removed using an electric razor and a depilatory cream, and the mice were fasted overnight. Cy5-labelled PUA or RPUA was administrated via intraperitoneal (i.p.) injection at a dose of 11 mg kg^{-1} body weight. 1 h after probe administration, mice received an i.p. injection of FAC at a concentration of 2.9×10^{-4} mg kg^{-1} ($n = 4$). Subsequently, the mice were anaesthetized with 10% isoflurane, and the whole-body images were captured at 30, 60, 90, 120, 150 and 180 min using an IVIS imaging system, with

wavelengths set for PUA ($\lambda_{ex}/\lambda_{em}$ = 520/790 nm), RPUA ($\lambda_{ex}/\lambda_{em}$ = 480/520 nm) and Cy5 ($\lambda_{ex}/\lambda_{em}$ = 650/670 nm). After image acquisition, the mice were sacrificed, and major tissues, including the heart, liver, spleen, lungs, and kidneys, were resected immediately for imaging. Semi-quantitative analysis of Cy5 and polymer signals were conducted using ImageJ software. Tissue sections from each group were imaged by CLSM. Results from the IVIS systems were analyzed with Living Image 4·4 (Caliper LS), with fluorescence signal corrected by subtracting background autofluorescence.

In vitro cytotoxicity assay

The 3T3 cells were incubated with PUA or RPUA at various concentrations in 96-well plates for 72 h. Cell viability was assessed using the cell counting kit-8 (CCK-8), following the manufacturer's instructions. After the addition of CCK-8 reagent, the cells were incubated for an additional 2 h. The absorbance at 460 nm was then measured using a microplate reader (Varioskan LUX). Cytotoxicity was expressed as the relative percentage of the cell viability compared to the control group.

Iron assay

Total iron content in cells and tissues was determined using a commercially available iron colorimetric assay kit. Cells or tissues were lysed with radio immunoprecipitation assay lysis buffer for 4 h at room temperature. The lysates were then centrifuged at 10,000 rpm for 15 min. The supernatants were collected and then mixed with the detection reagent provided in the kit and incubated at 37 °C for 30 min. The reaction mixtures were subsequently transferred to a 96-well plate, and the absorbance at 562 nm was recorded using a microplate reader. A standard curve was generated in parallel using the supplied iron standard and the same experimental procedure. Total iron content was calculated according to the standard curve.

Cell viability assays

Cell viability was determined with the CCK-8 method. Briefly, MCF-7 cells were seeded at 5×10^3 cells/well in 96-well plates and cultured overnight for subsequent experiments. To detect cytotoxicity, polymer-treated MCF-7 cells were incubated with FAC (10 μM) for 24 h. Then, the incubation solution was removed and a CCK-8 working solution was added for 30 min, and the absorbance was detected at 450 nm with a multimode microplate reader (EnSpire, PerkinElmer, USA).

In vivo acute and chronic toxicity evaluation

Healthy mice were fasted overnight and intraperitoneally injected with Cy5-labelled PUA at a dose of 11 mg kg^{-1}. Saline was used as the control. 3 h post-treatment, the mice were euthanized, and their major organs (liver, spleen, kidney, heart, and lung) were collected for histopathological analysis. Tissue sections (5 μm) were prepared and stained with hematoxylin and eosin (H&E). The stained sections were then subjected to both gross and microscopic pathological evaluations. To evaluate the chronic toxicity of PUA, healthy C57BL/6 mice were randomly assigned to two groups (n = 4). One group was intraperitoneally injected with PUA (200 μL, 2 mg mL^{-1}) once a day for 10 consecutive days, while the other group received an equivalent volume of saline (200 μL) as a control. The body weight of the mice was monitored daily throughout the experiment. On day 10, the mice were euthanized, and blood samples were collected from the ophthalmic artery. The blood samples were analyzed for routine hematological parameters and hepatic and renal function tests. Major organs (liver, spleen, kidney, heart, and lung) were surgically excised, fixed in 10% formalin for 24 h, and embedded in paraffin. Tissue sections (5 μm) were stained with hematoxylin and eosin (H&E) and subjected to gross and microscopic pathological evaluations for histopathological analysis.

Statistical analysis

The obtained quantitative data were expressed as means ± standard deviations (SD). Statistical analyses were performed as described in the corresponding figure legends. Statistical analysis was performed using one-way ANOVA for comparisons among multiple groups or the two-tailed paired Student's t-test for comparisons between two groups. All statistical analyses were performed using GraphPad Prism software, version 8.0.2. Statistical significance was defined as $^{*}p<0.05$, $^{**}p<0.01$, $^{***}p<0.001$, and $^{****}p<0.0001$.

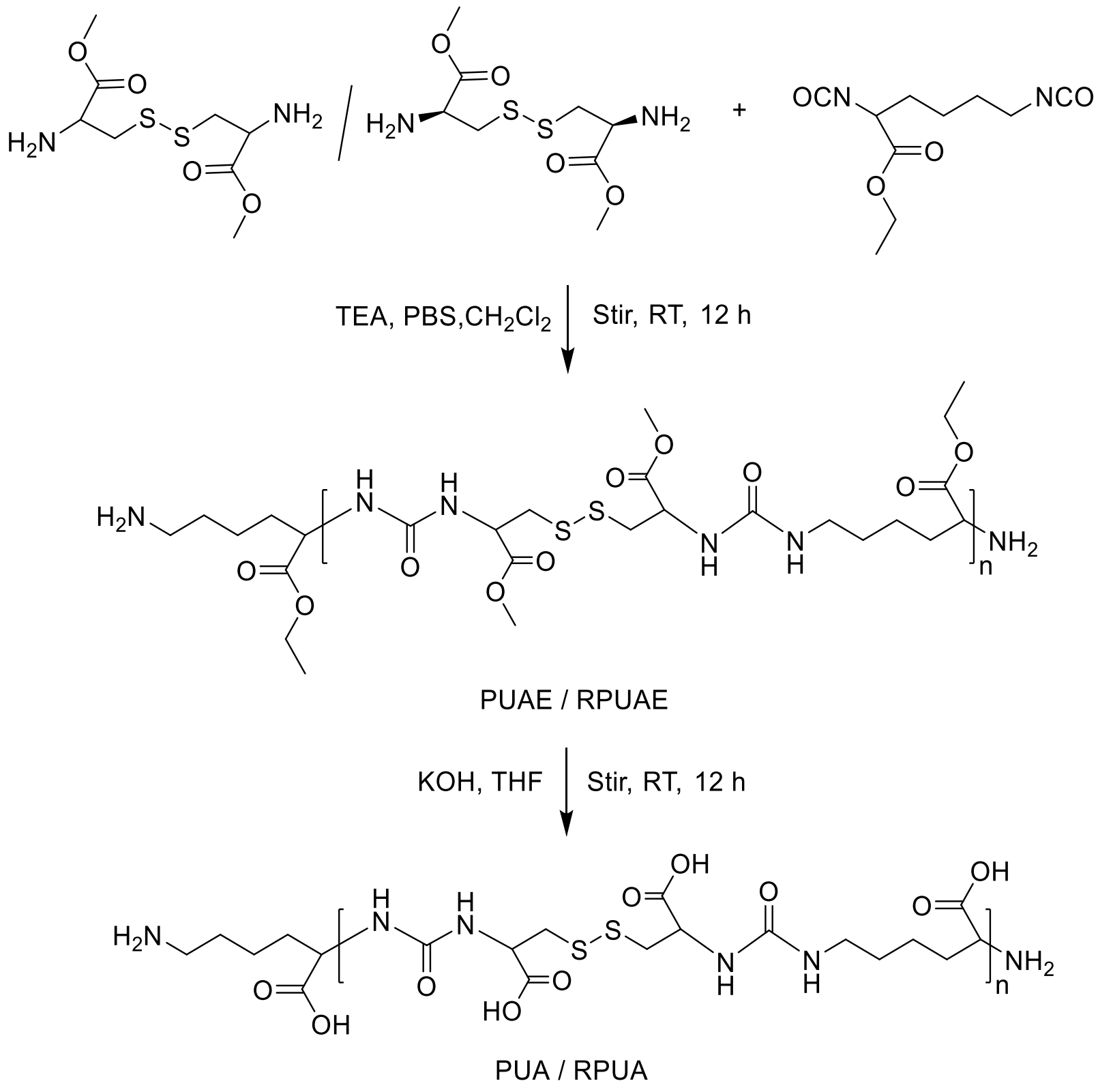

Figure S1. Synthesis of PUA and RPUA (70-80% yield).

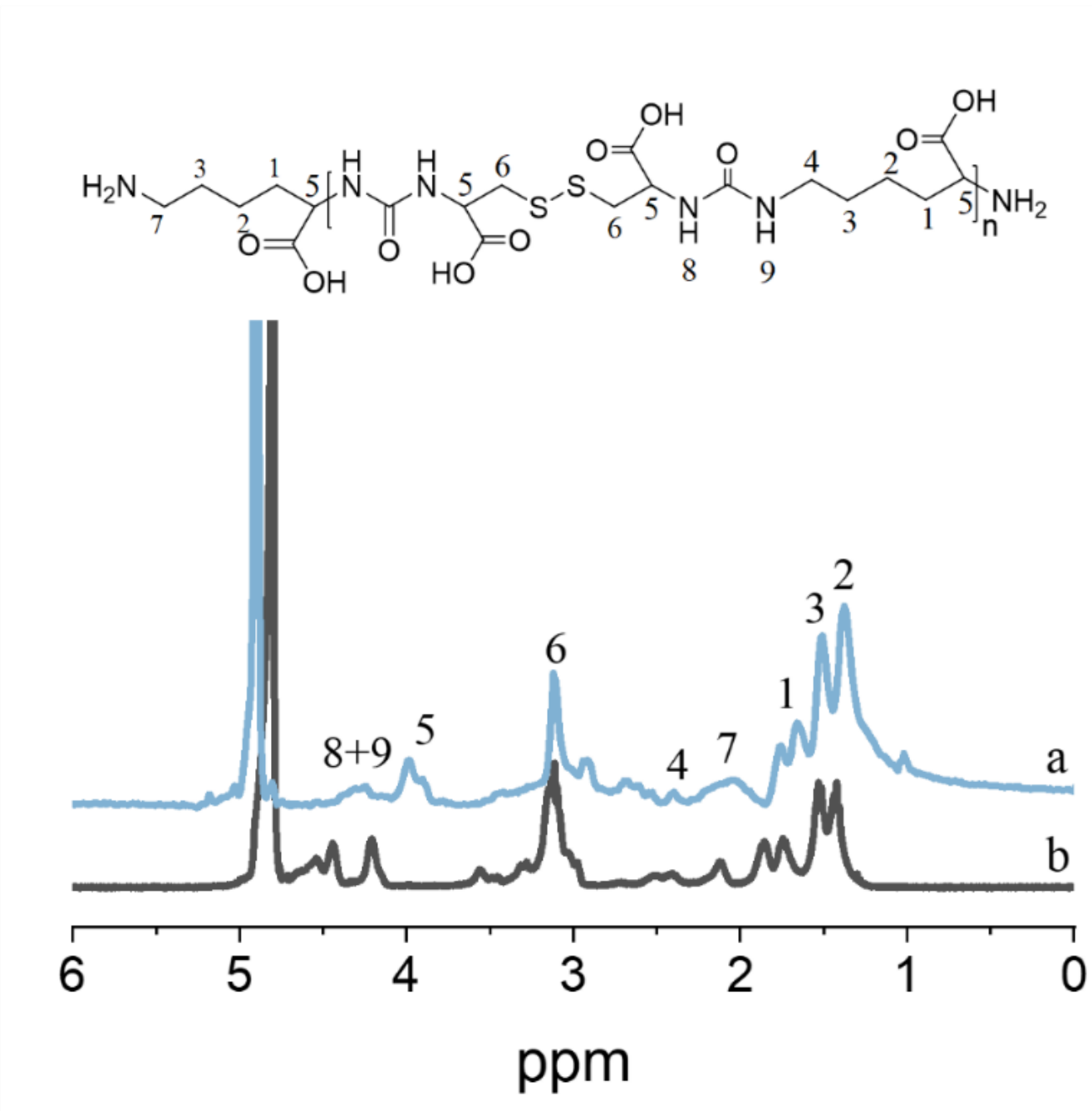

Figure S2. ^{1}H NMR spectra of RPUA (a), PUA (b) in D_2O.

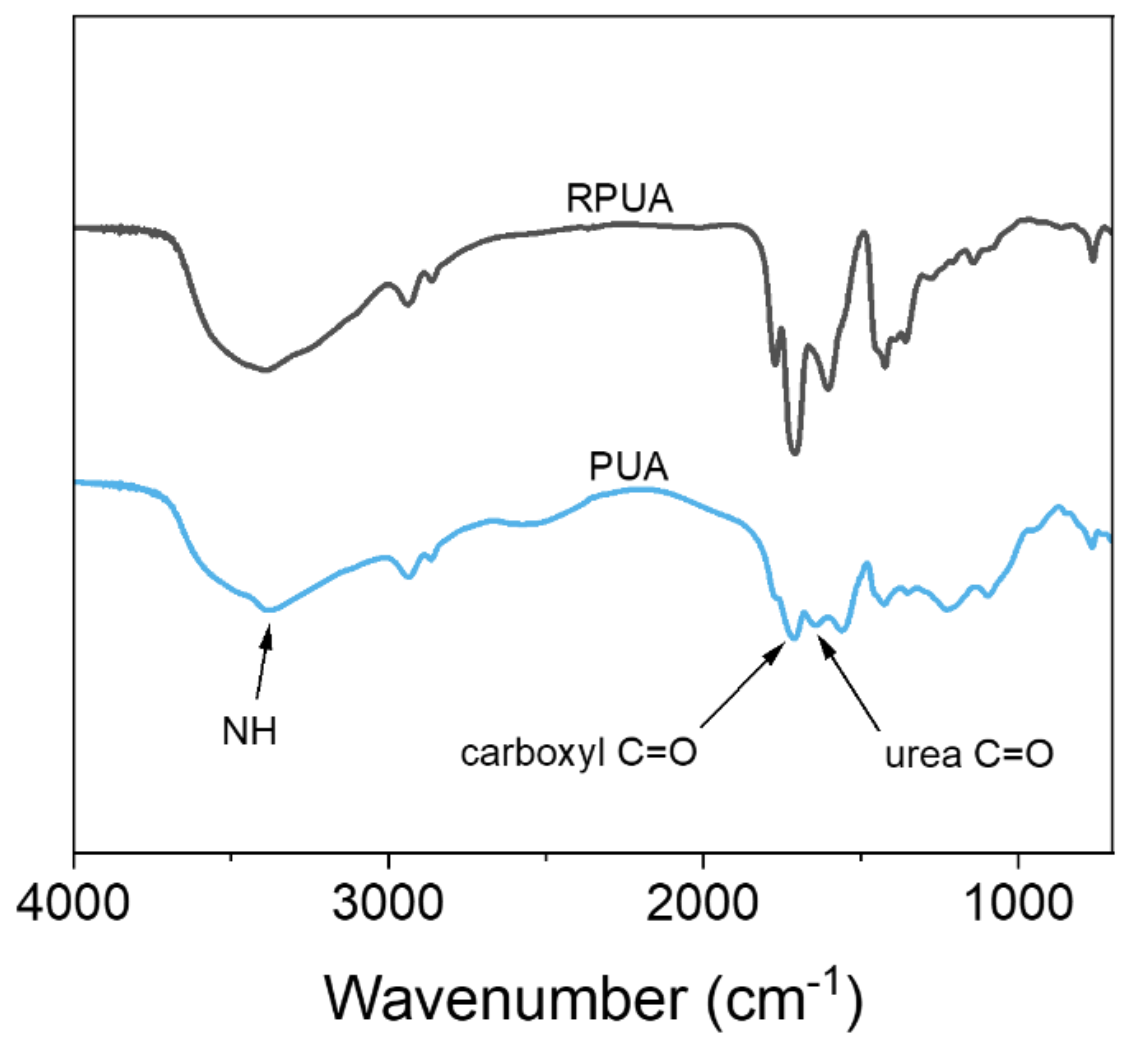

Figure S3. FTIR spectra of PUA and RPUA.

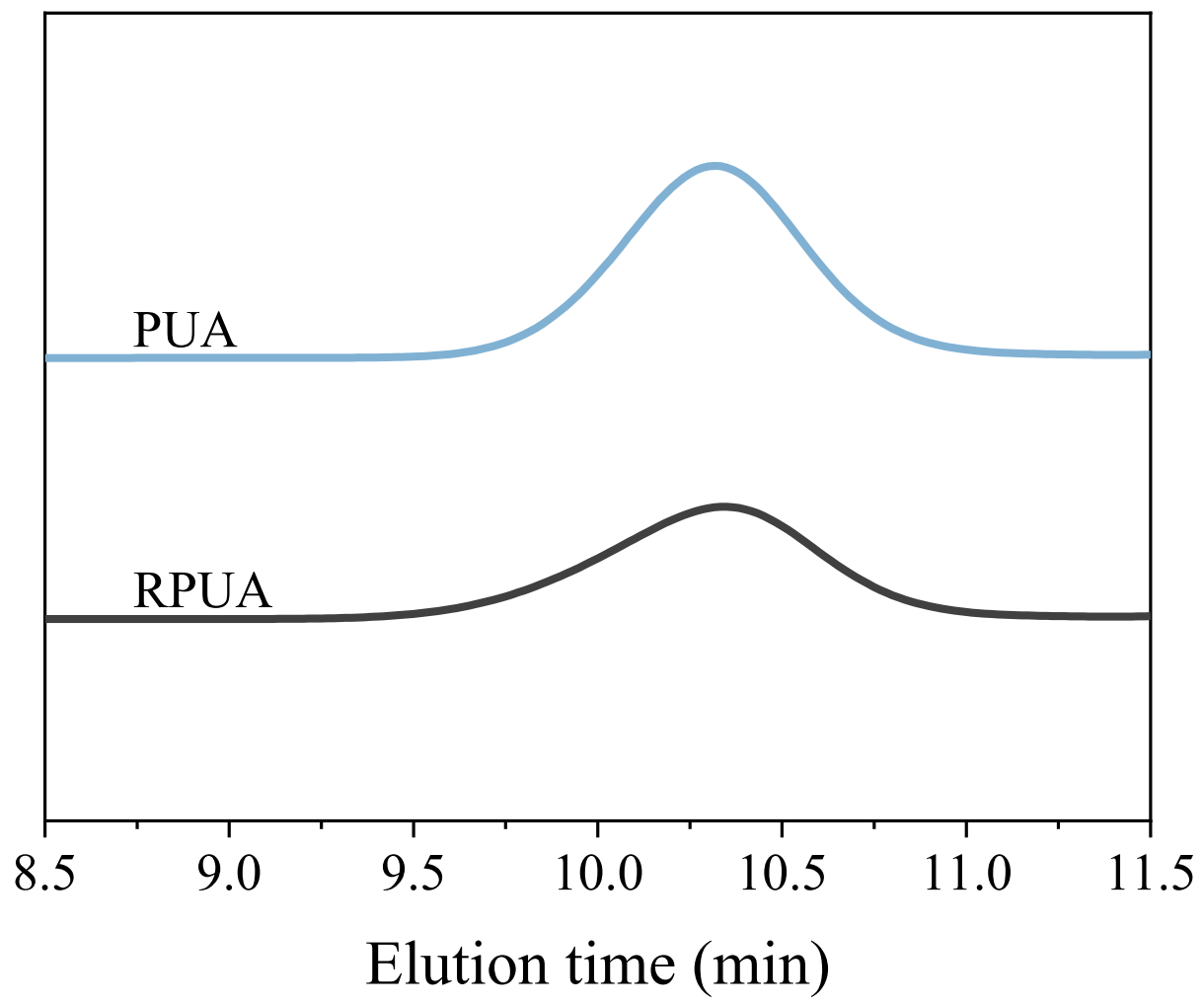

Figure S4. GPC curves of PUA and RPUA.

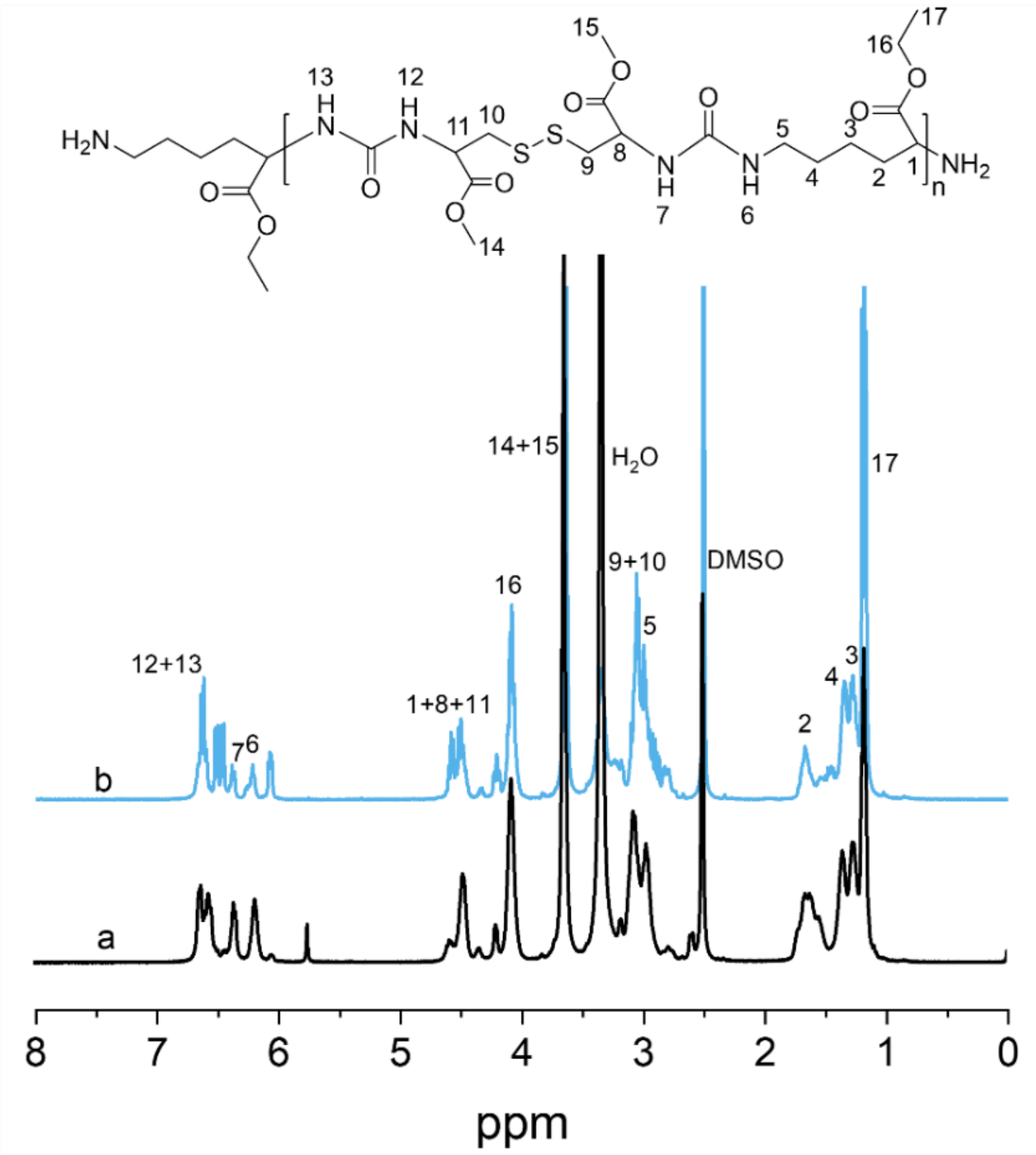

Figure S5. ^{1}H NMR spectra of PUAE and RPUAE.

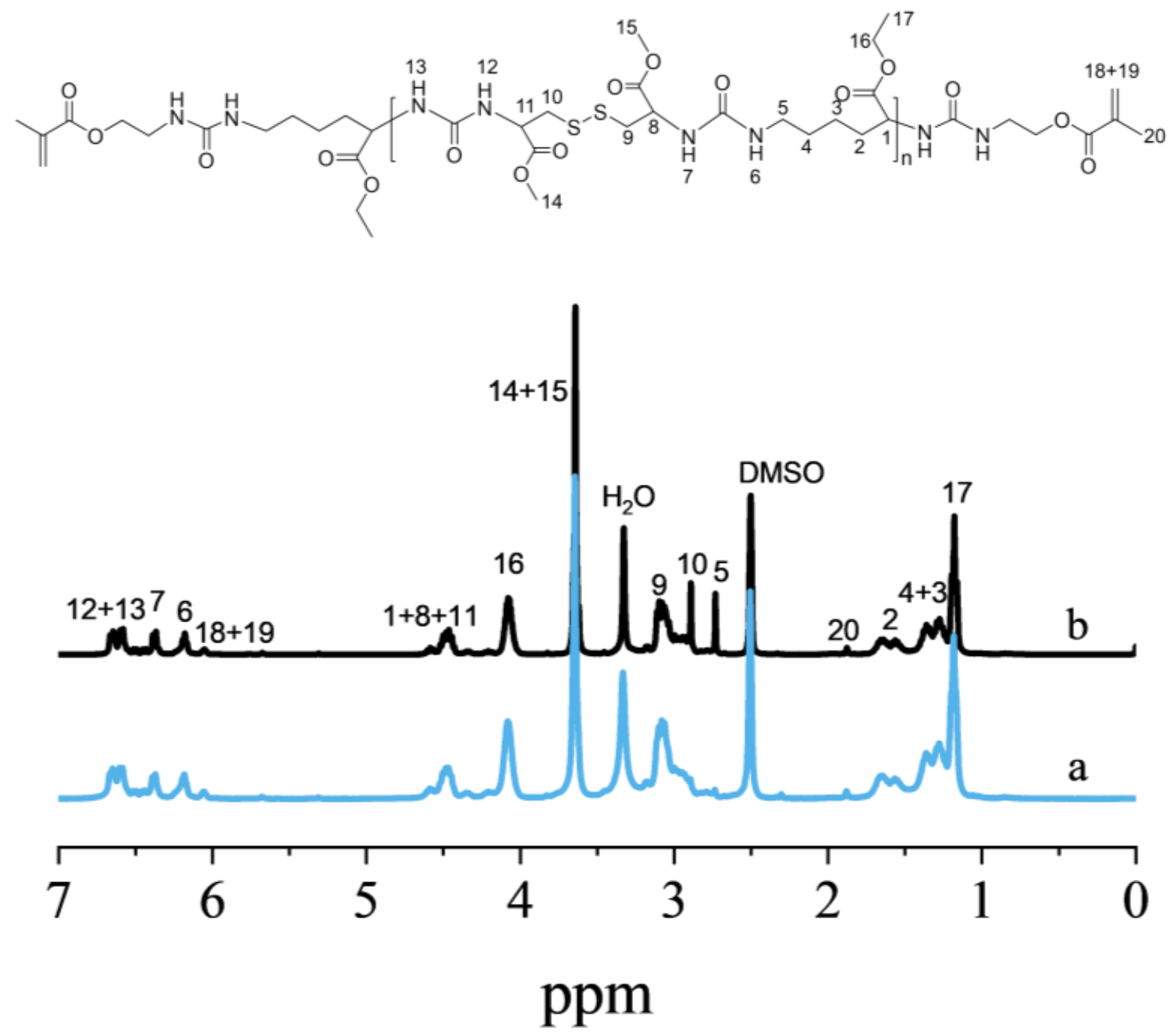

Figure S6. ^{1}H NMR spectra of IEM-labeled PUAE and RPUAE.

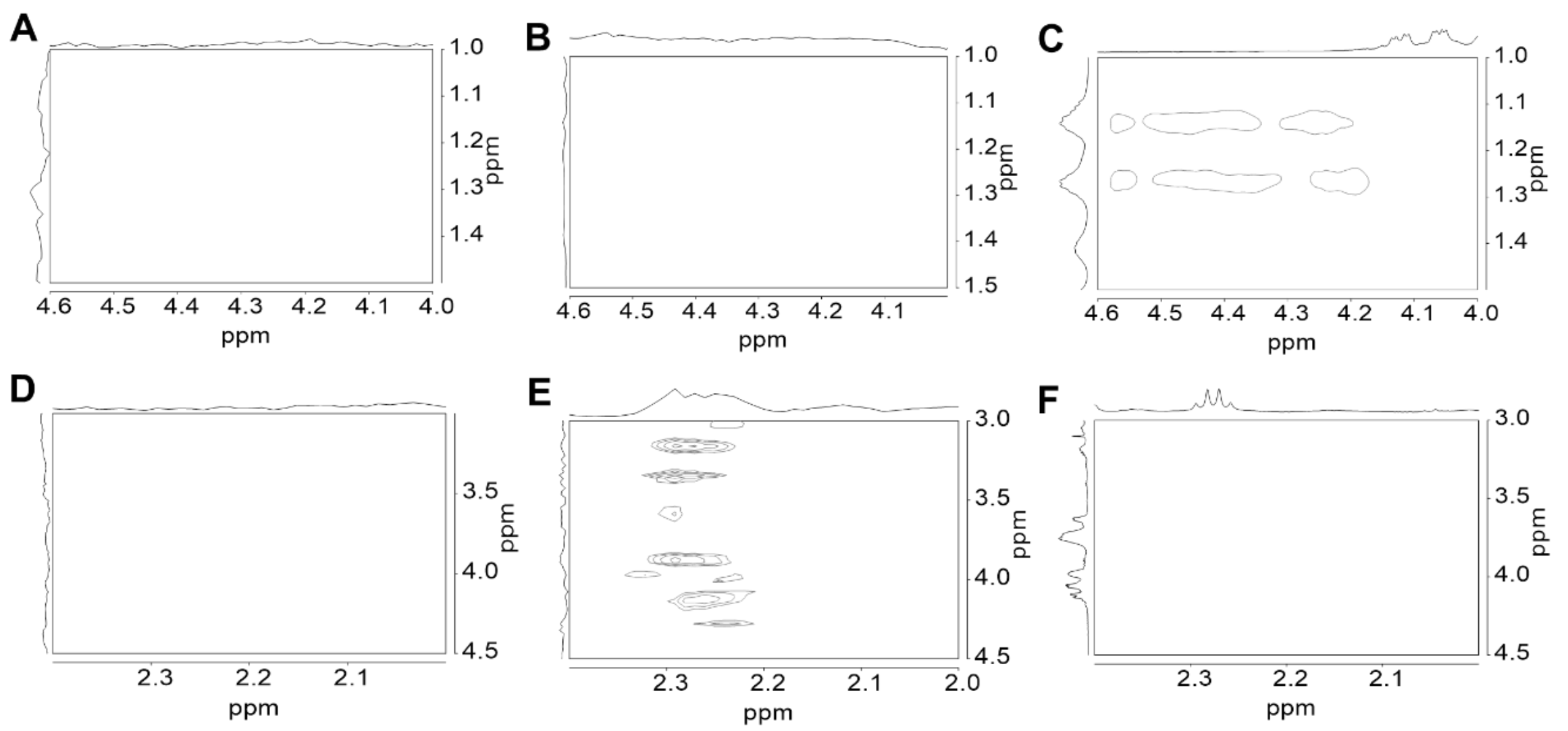

Figure S7. ^{1}H-^{1}H NOESY spectra containing major NOEs of RPUA (**A**, **D**), PUA (**B**, **E**) and PUA at basic pH (**C**, **F**), recorded in D_2O.

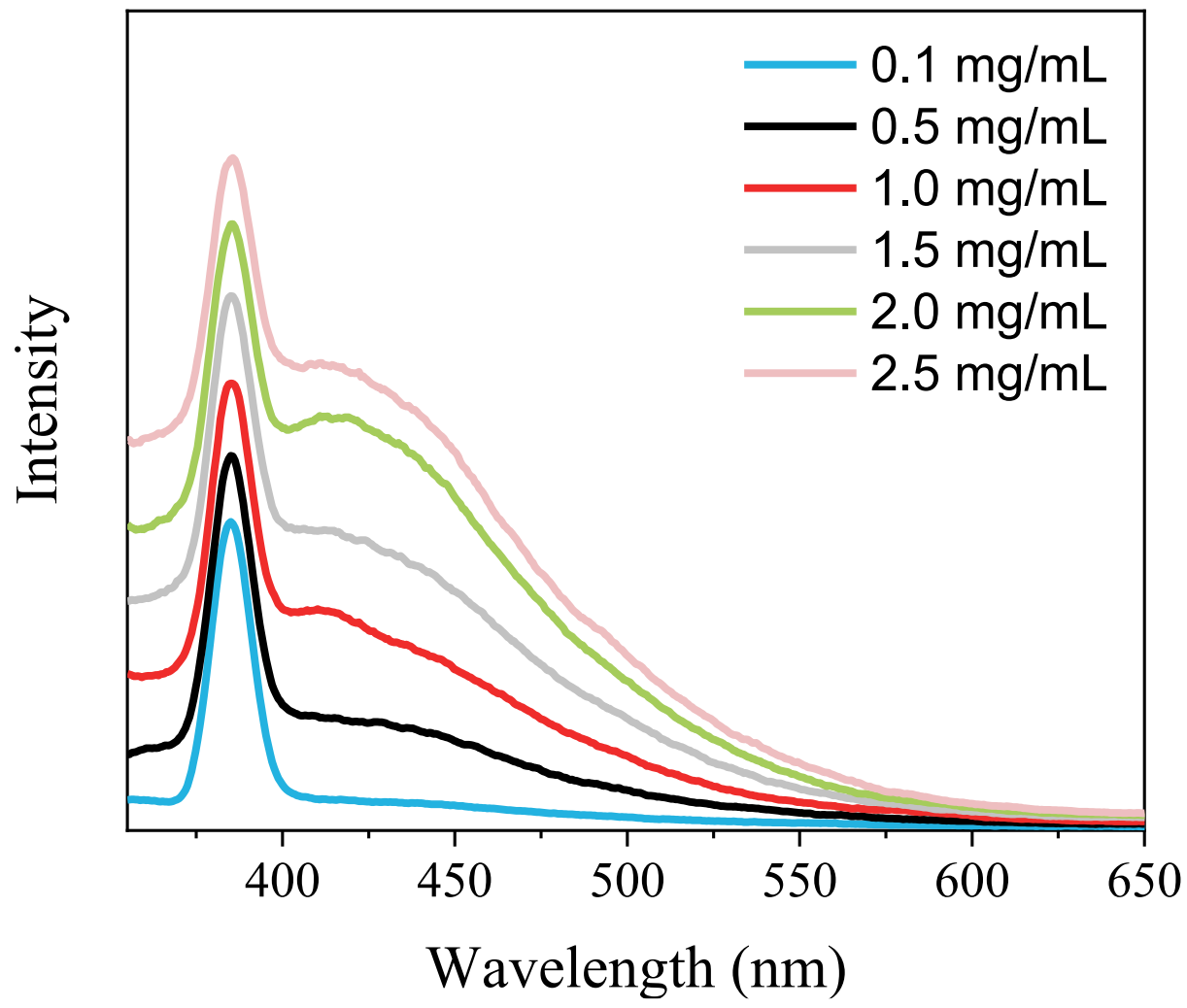

Figure S8. Fluorescence spectra of PUA aqueous solutions with different concentrations. λ_{ex} = 340 nm.

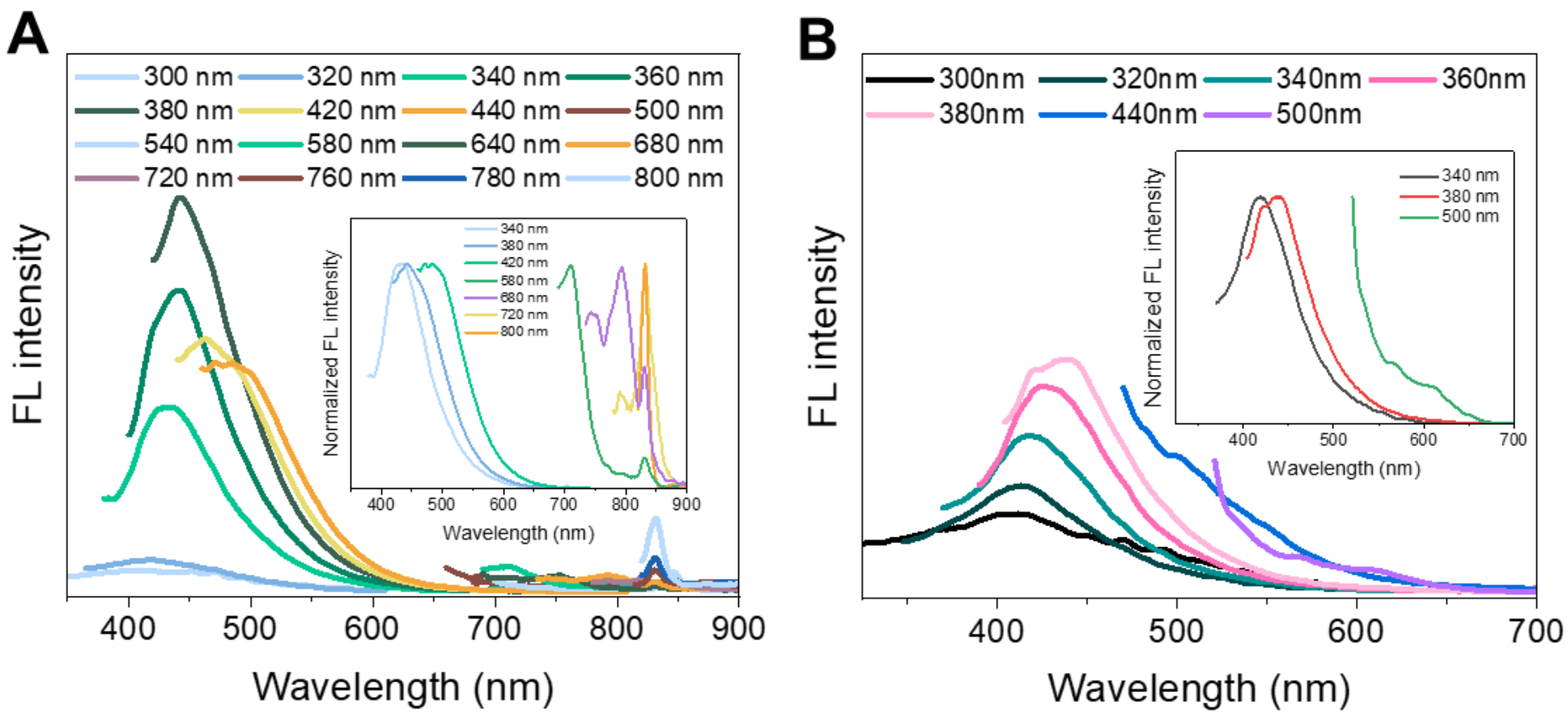

Figure S9. Fluorescence spectra of PUA (**A**) and RPUAE (**B**) at different λ_{ex}, inset represents normalized fluorescence spectra.

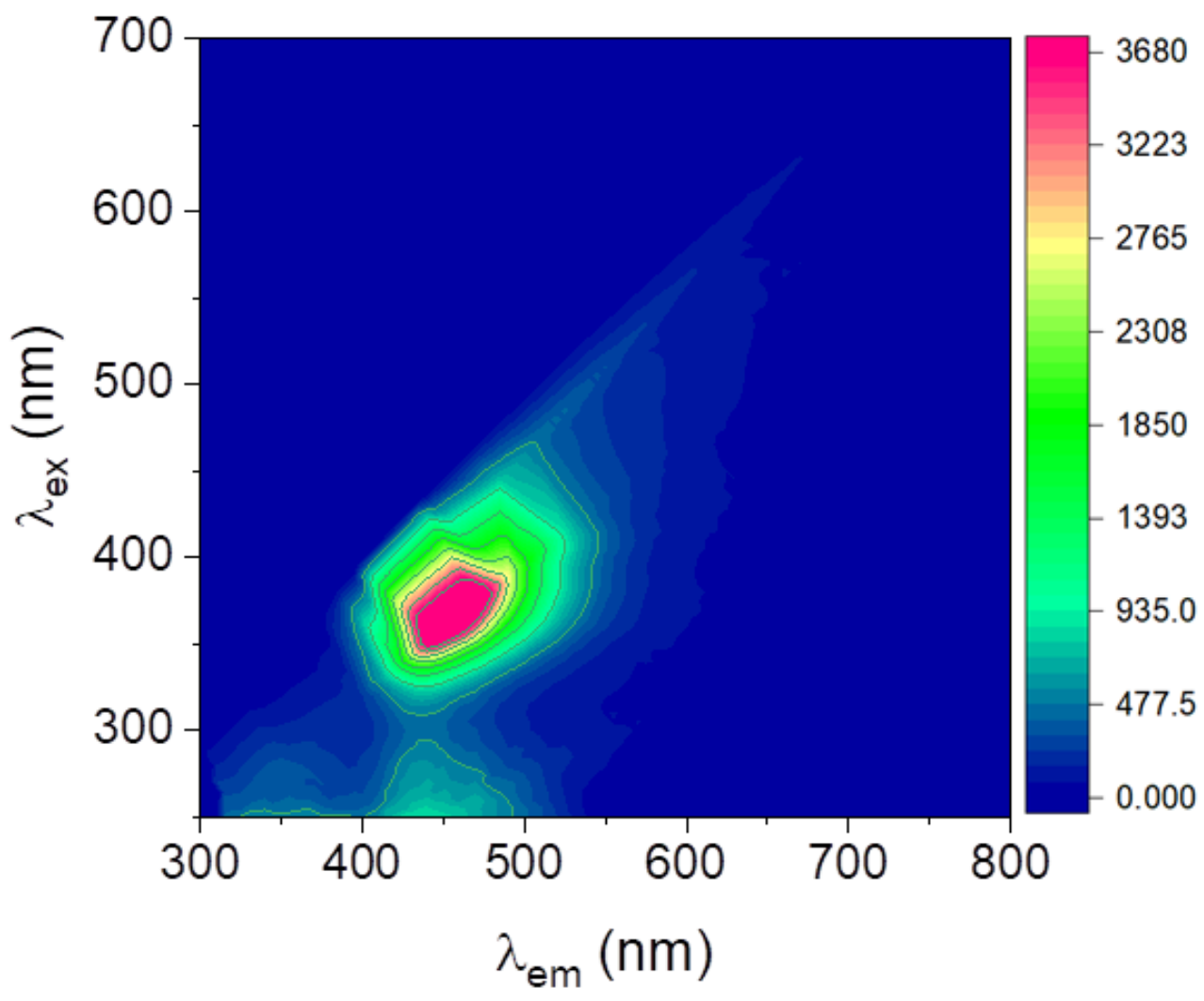

Figure S10. 3D fluorescence spectrum image of PUA film.

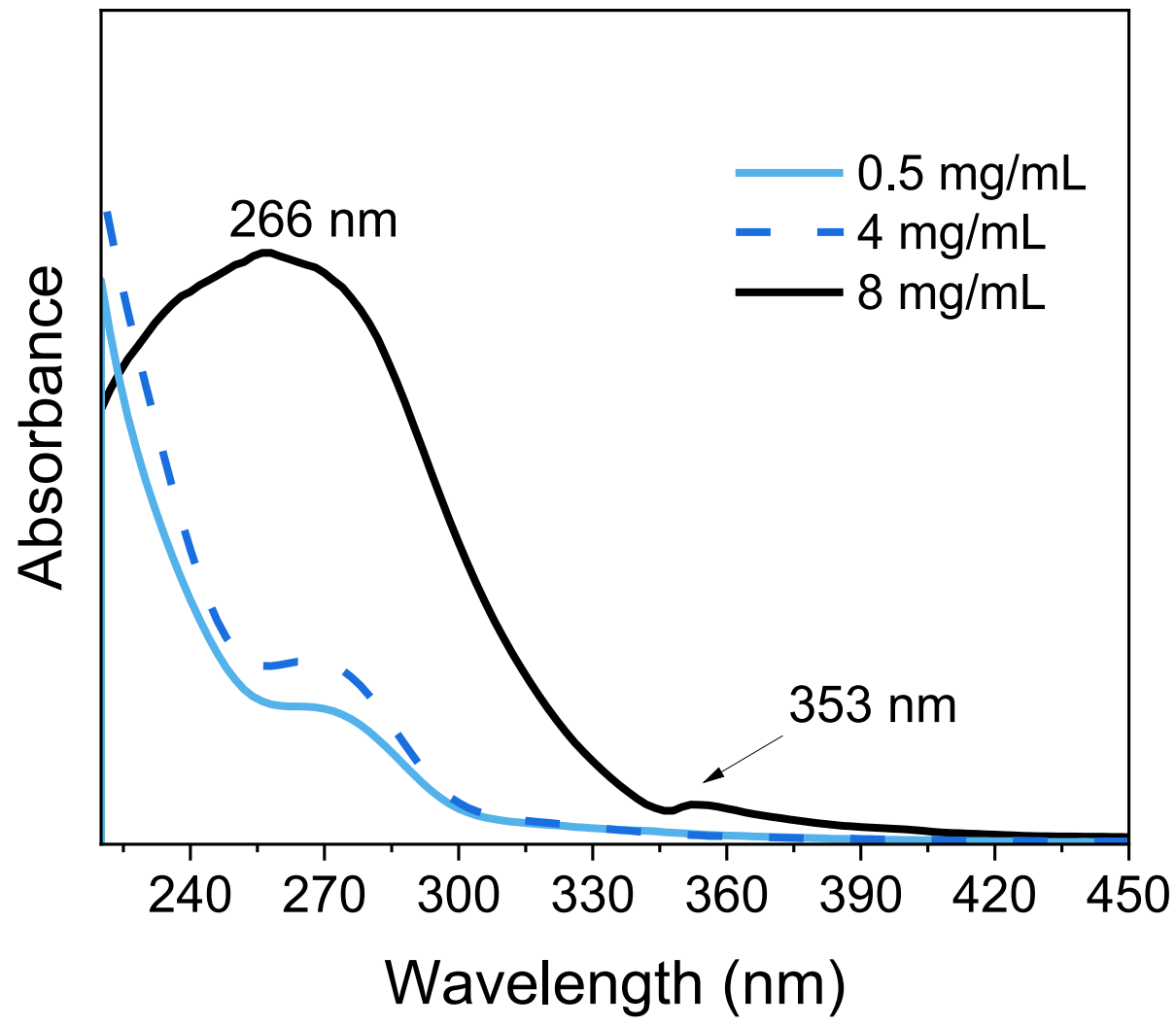

Figure S11. Excitation spectra of PUA in aqueous solutions of varying concentrations were measured at an emission maximum of 440 nm.

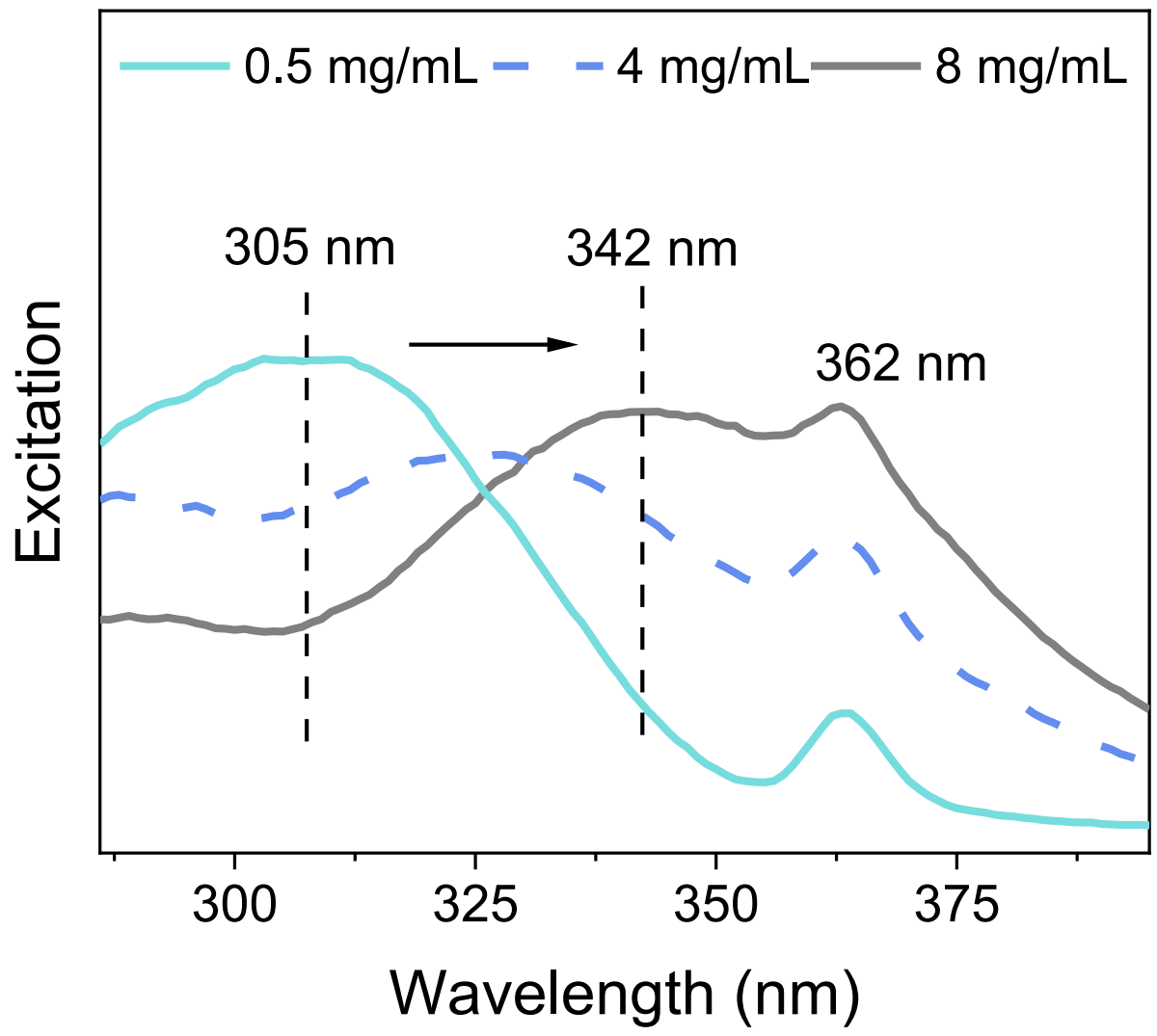

Figure S12. Concentration-dependent excitation spectra of PUA aqueous solutions for emission at 440 nm.

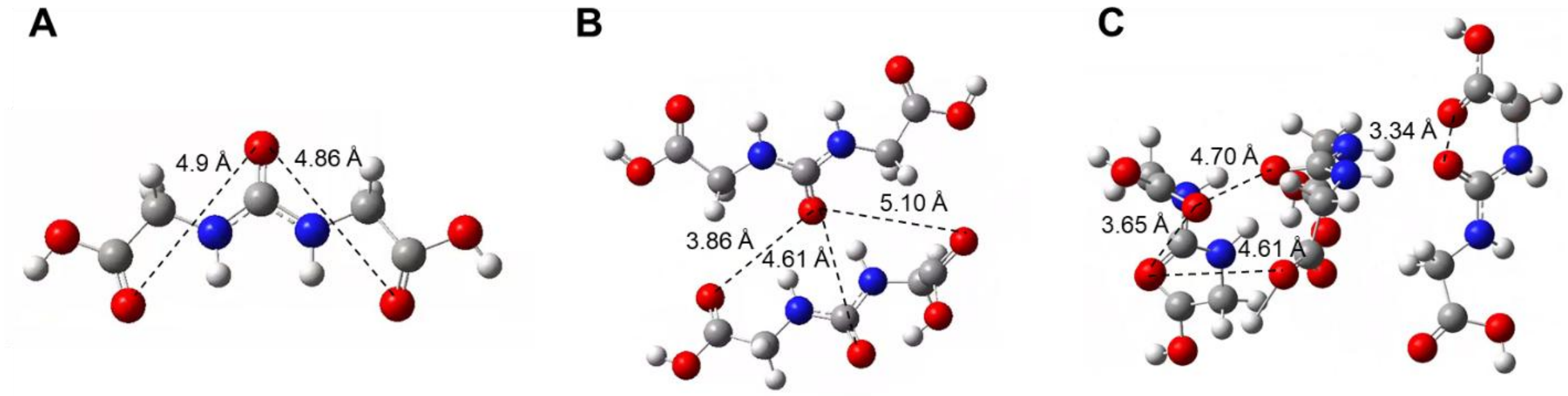

Figure S13. Mechanism of multicolor emission based on theoretical calculations. Optimized excited state geometries of clustering models are presented to elucidate the mechanism of multicolor emission. Black dotted lines represent the interchain or intrachain distance ($d_{O\cdots C=O}$) involving the carbonyl group (white: H; grey: C; red: O; blue: N). The molecules progressively aggregated from monomolecular (**A**) to dimolecular (**B**) and trimolecular (**C**), leading to a decrease in the distances between carbonyl groups, from 4.88 to 4.07 Å, resulting in a red shift and enhanced emission in the optimal λ_{em} for polymeric aggregates.

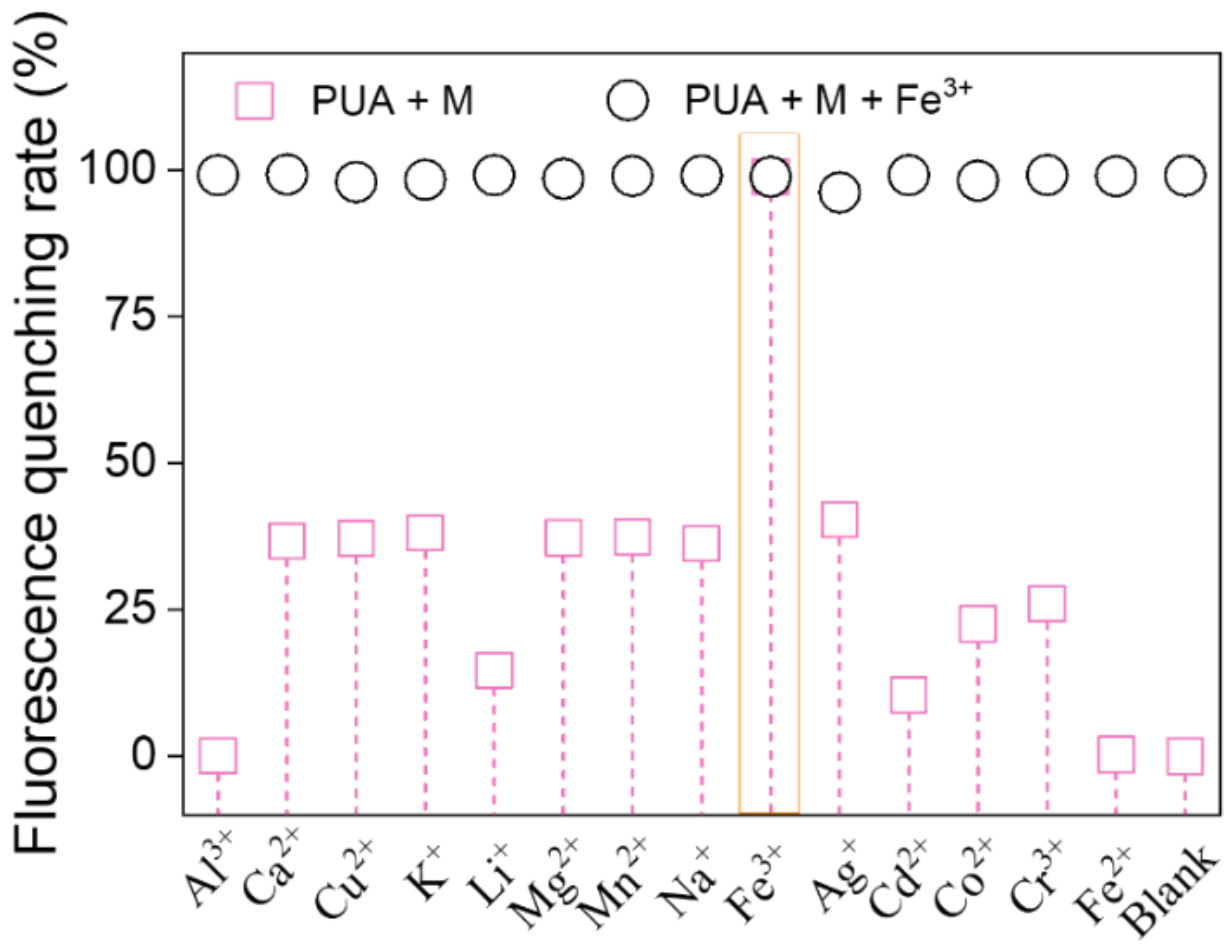

Figure S14. Fluorescence quenching rate of PUA in response to various mental ions.

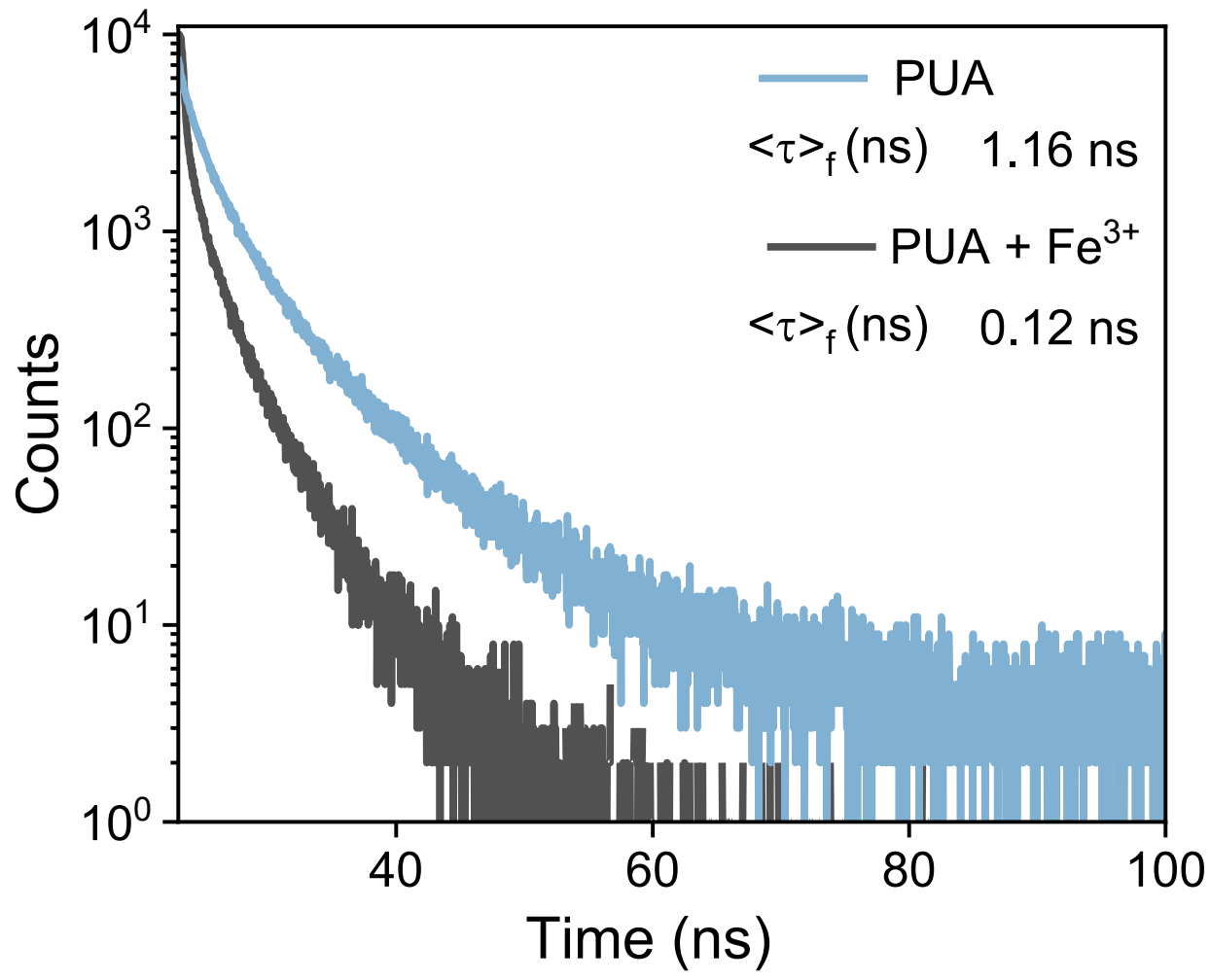

Figure S15. Lifetime decay profiles of PUA in the absence and presence of Fe^{3+} (λ_{ex} = 370 nm).

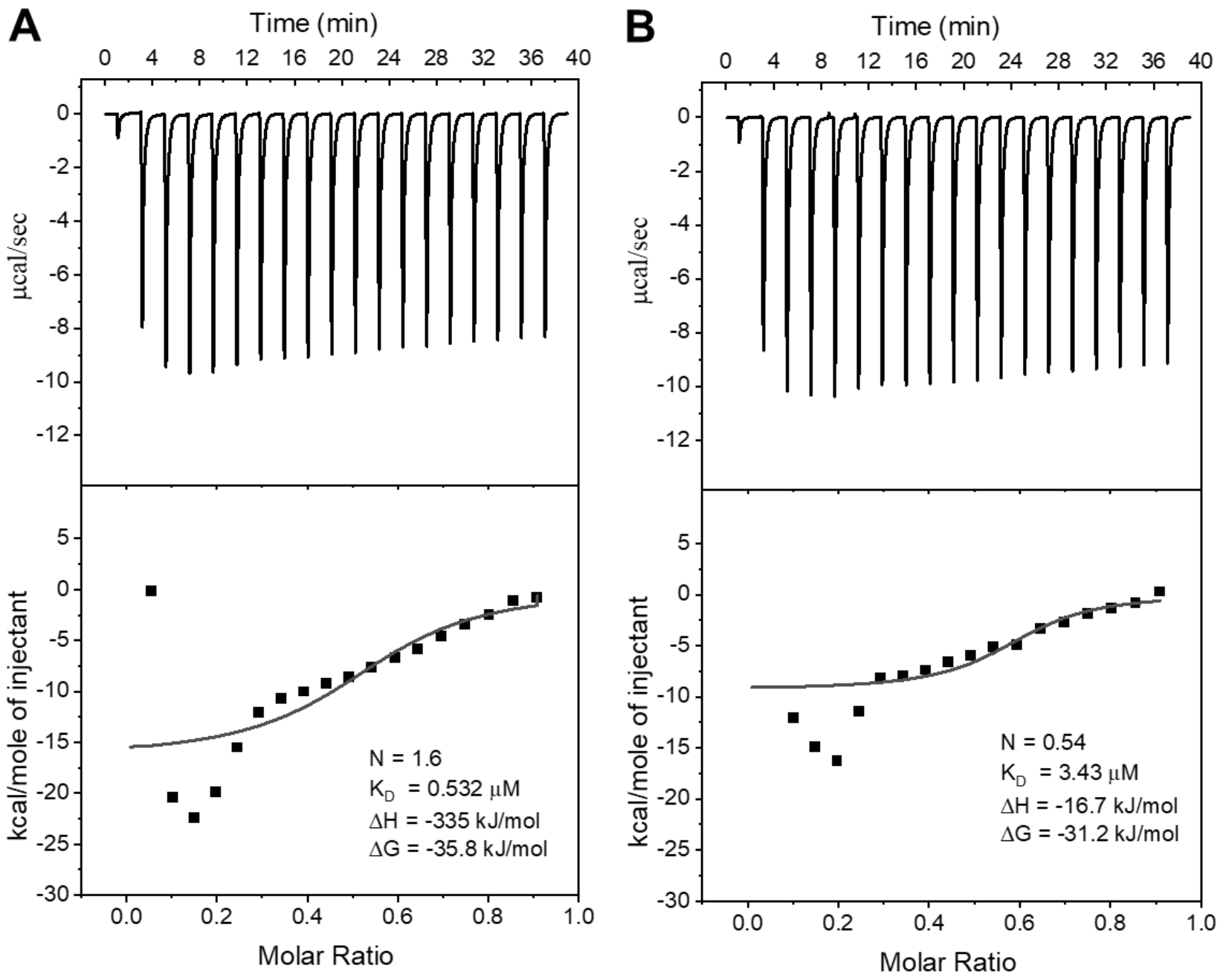

Figure S16. ITC measurement of the representative Fe^{3+}-binding PUA (**A**) and RPUA (**B**).

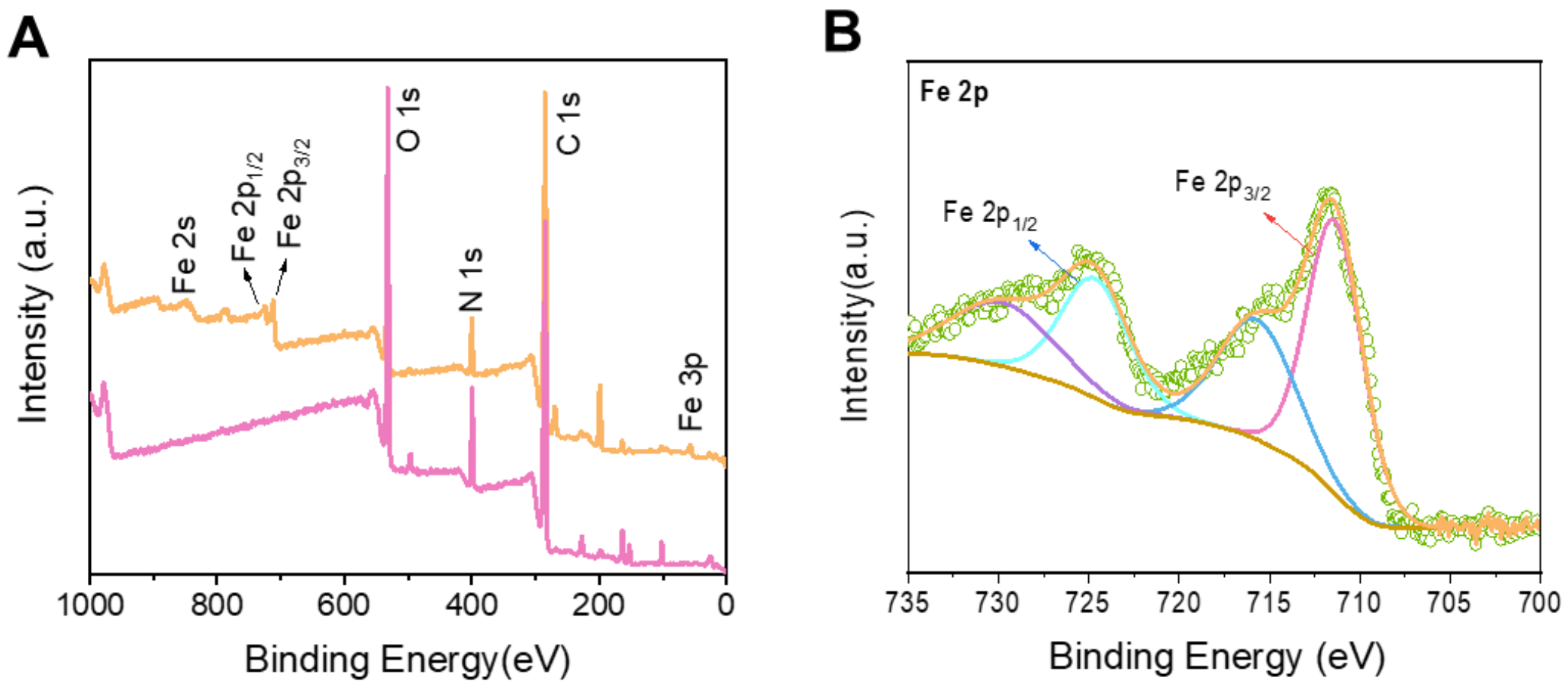

Figure S17. Additional XPS analysis of PUA and PUA-Fe^{3+}. (**A**) Survey spectra of PUA and PUA-Fe^{3+}. (**B**) High-resolution XPS spectra of Fe 2p for PUA-Fe^{3+}, including satellite peaks. The signals corresponding to Fe 2p, C 1s, O 1s, and N 1s are observed in the overview spectrum of PUA-Fe^{3+}.

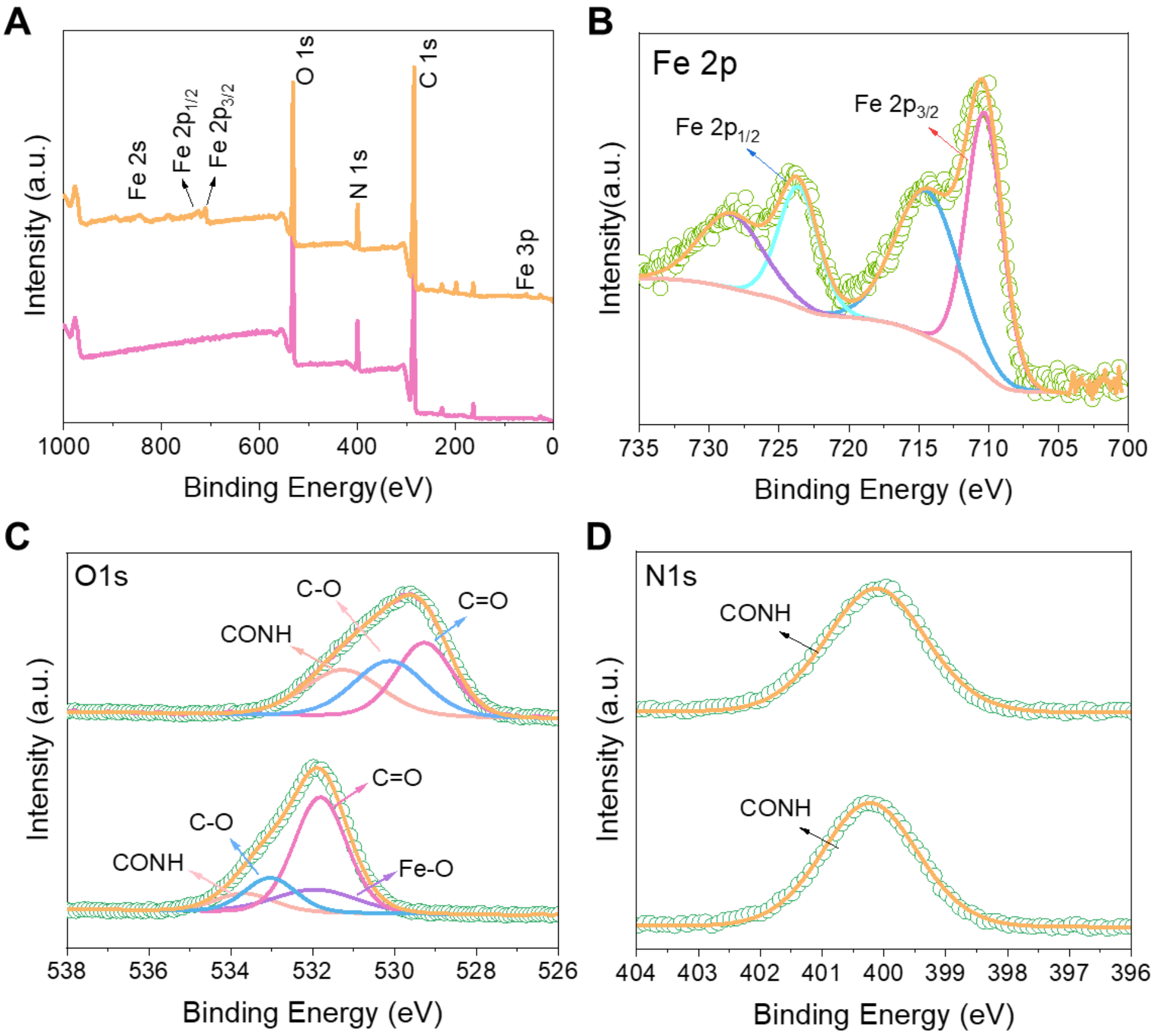

Figure S18. XPS analysis of RPUA and RPUA-Fe^{3+}. (**A**) Survey spectra of PUA and PUA-Fe^{3+}. (**B**) High-resolution XPS spectra of Fe 2p for RPUA-Fe^{3+}, including satellite peaks. (**C**, **D**) High-resolution XPS spectra of O 1s (C), and N 1s (D) for RPUA and RPUA-Fe^{3+}. The signals corresponding to Fe 2p, C 1s, O 1s, and N 1s are observed in the overview spectrum of RPUA-Fe^{3+}.

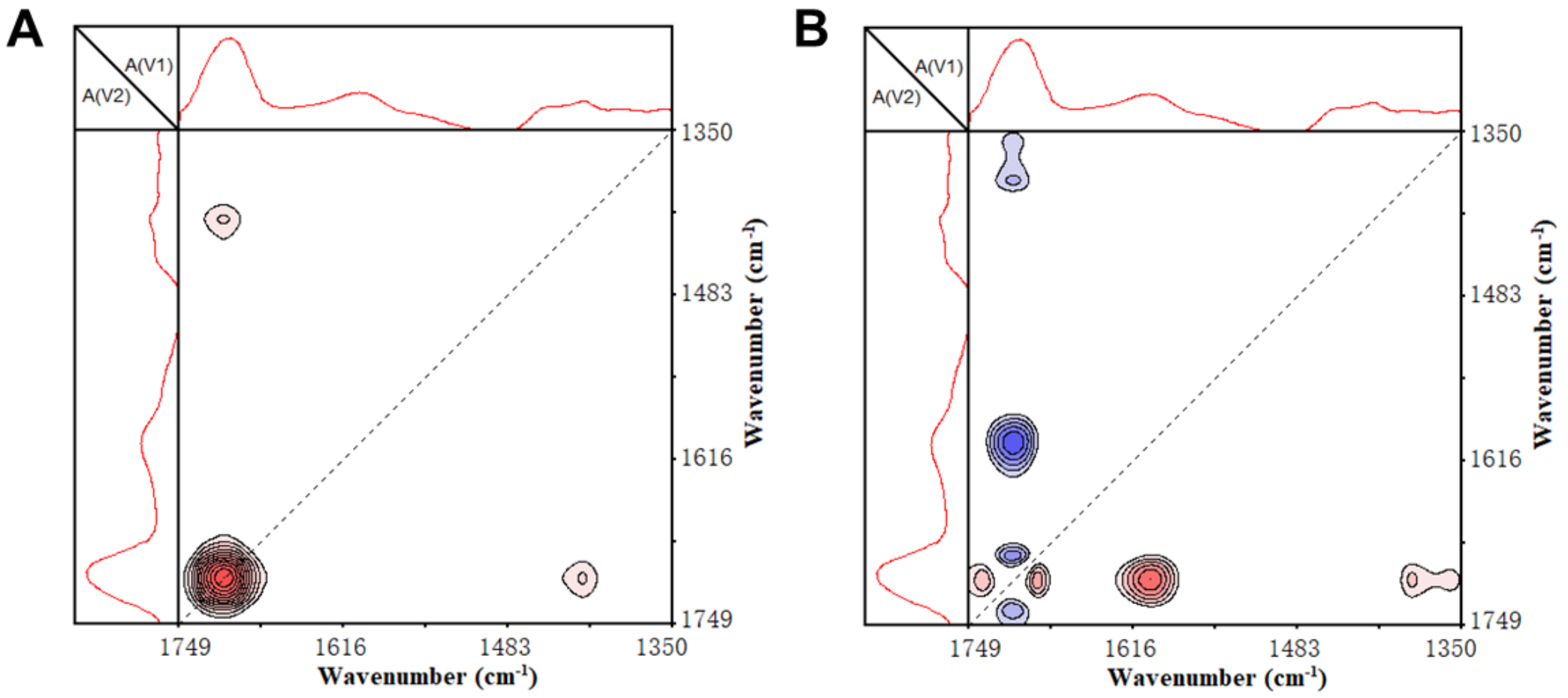

Figure S19. (**A**, **B**) Synchronous (A) and asynchronous (B) 2D correlation maps generated from the 1749-1350 cm^{-1} region of FTIR spectra of RPUA-Fe^{3+}. Red regions represent positive correlations, while blue regions represent negative correlations. A higher color intensity indicates a stronger correlation.

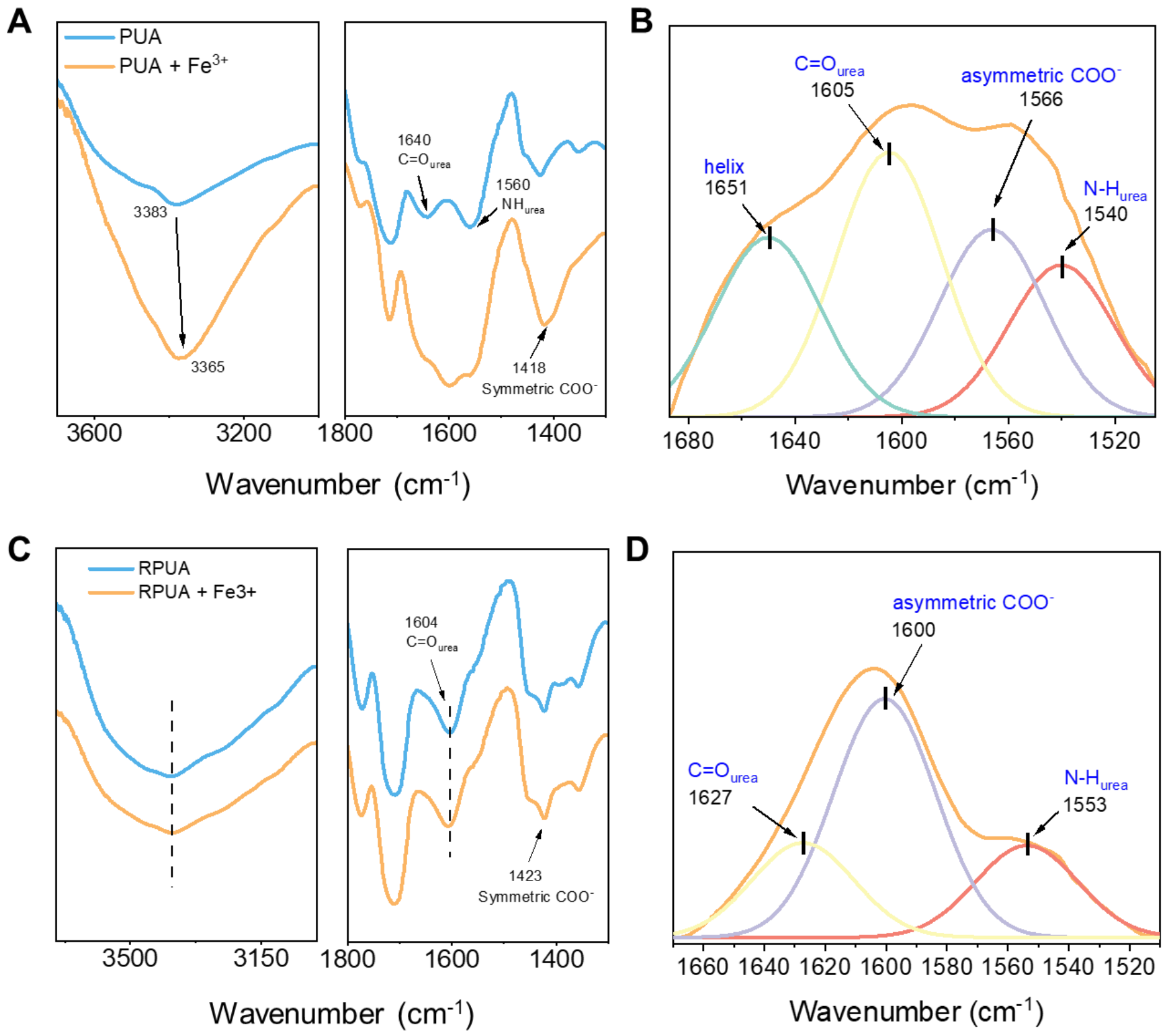

Figure S20. FTIR spectra of PUA (**A**) and RPUA (**C**) before and after Fe^{3+} coordination. Multi-peak fitting analysis of the FTIR spectra of PUA (**B**) and RPUA (**D**) after Fe^{3+} coordination, fitted with gaussian functions.

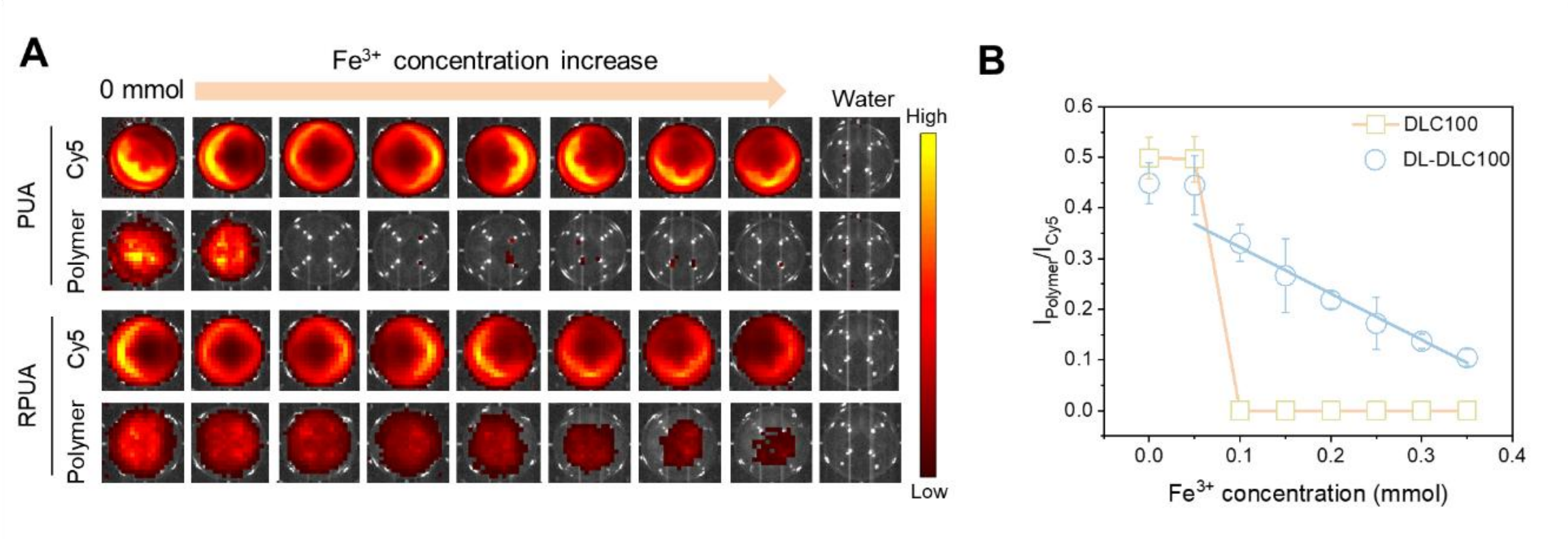

Figure S21. (**A**) In vitro luminescent images of well plates showing the intensities changes of Cy5-PUA aqueous solutions upon addition of different concentrations of Fe^{3+}. Luminescence imaging was performed using an IVIS Spectrum imaging system. (**B**) Calibration curves depicting the mean fluorescence intensity ratio $I_{polymer}/I_{Cy5}$ as a function of FAC aqueous solution concentrations. Data are presented as the mean ± standard deviation (SD).

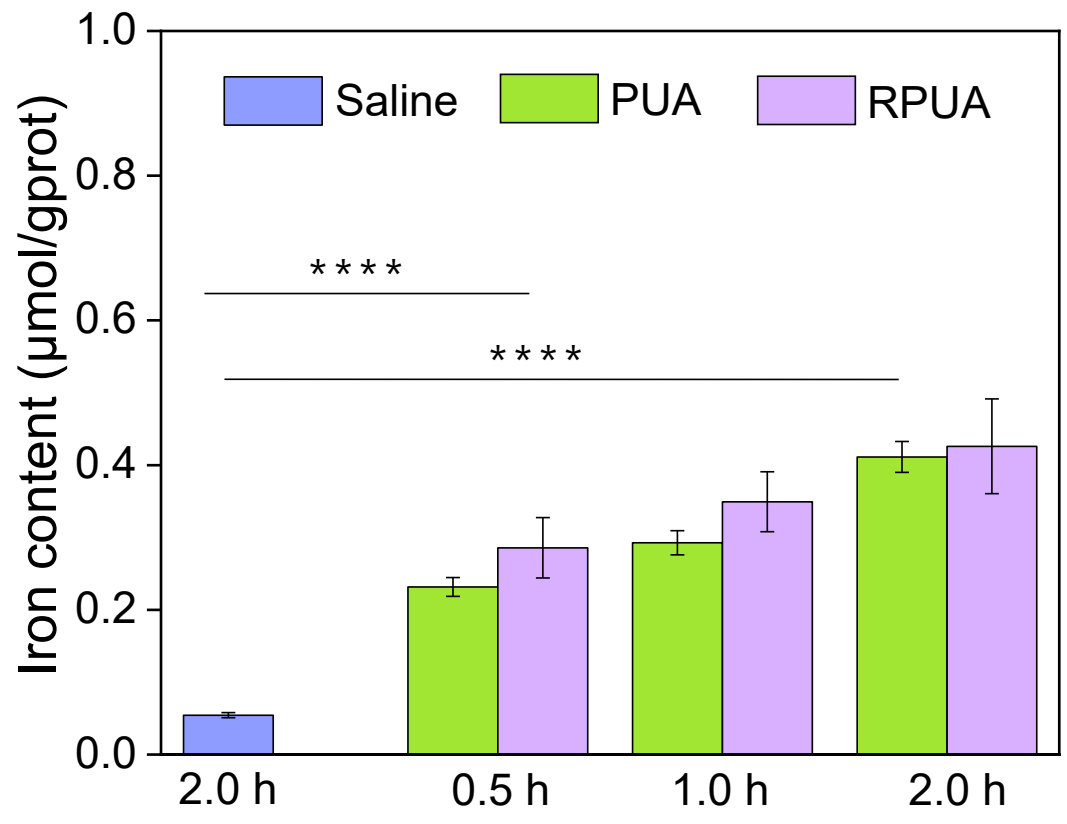

Figure S22. Quantification of the total iron content by an iron colorimetric assay kit on MCF-7 cells incubated with polymer for different times. Data are presented as mean ± standard deviation ($n = 3$ independent samples). Statistical significance was calculated by one-way ANOVA with Tukey's multiple comparisons test, ns: no significant; *: $p < 0.05$; **: $p < 0.01$; ***: $p < 0.001$; ****: $p < 0.0001$.

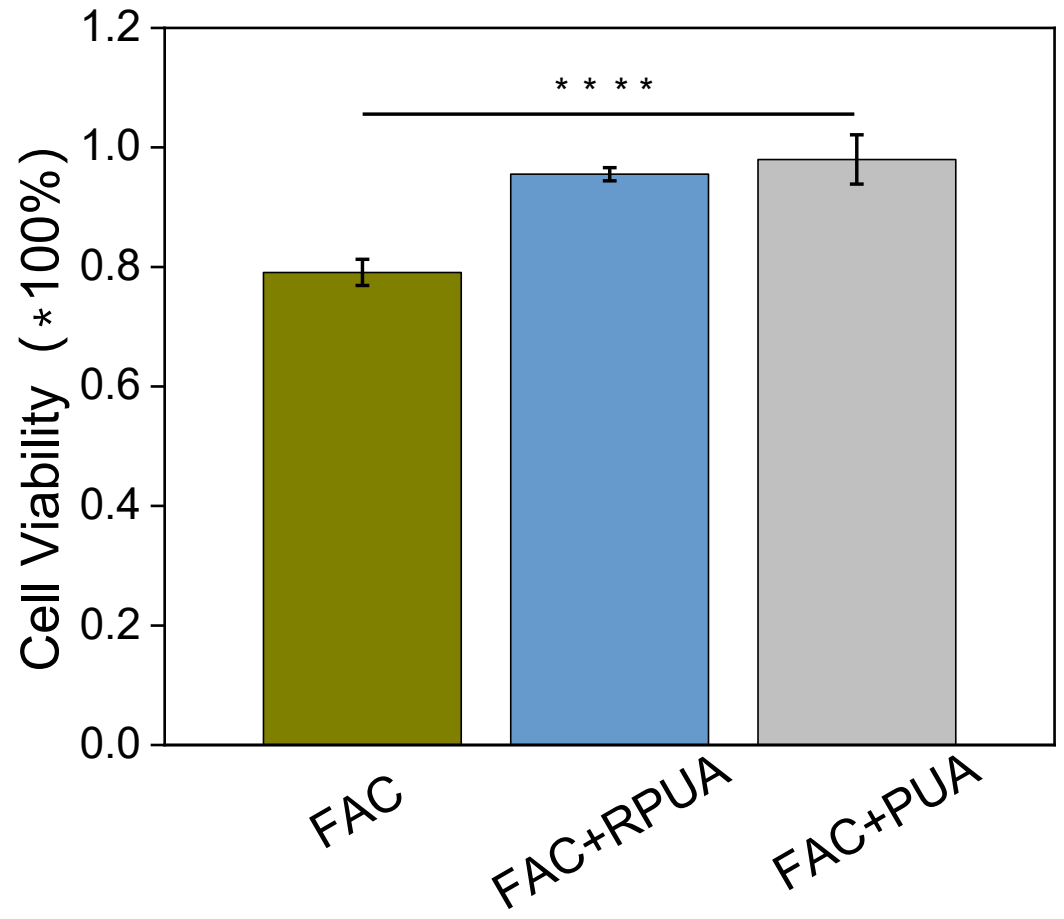

Figure S23. Polymer treatment rescued cell death in FAC treatment. Data are presented as mean ± standard deviation (n = 5 independent samples). Statistical significance was calculated by the two-tailed paired Student's t-test, ns: no significant; *: $p < 0.05$; **: $p < 0.01$; ***: $p < 0.001$; ****: $p < 0.0001$.

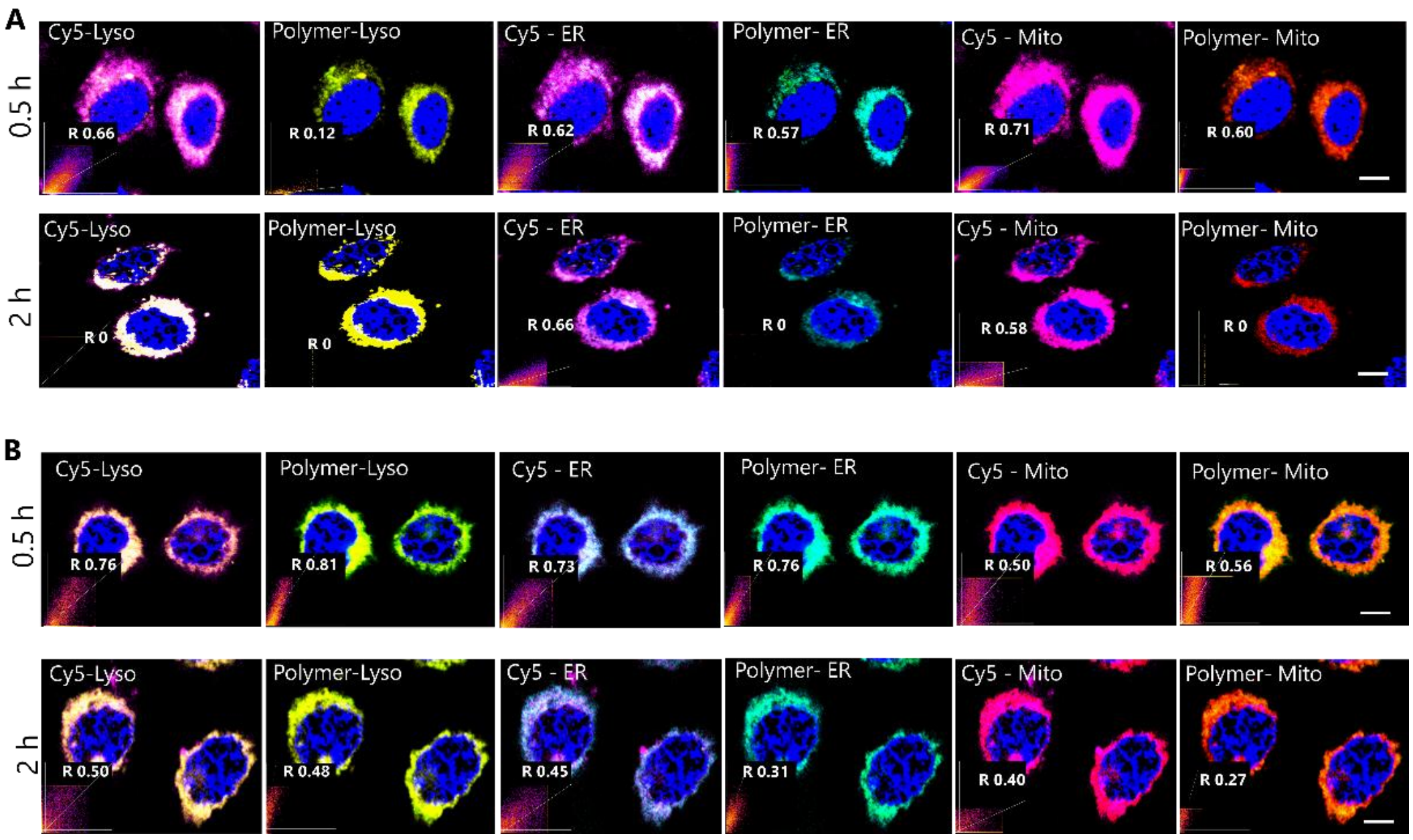

Figure S24. CLSM images of MCF-7 cells pretreated with Cy5-PUA (**A**) and Cy5-RPUA (**B**) (1 mg/mL) incubated with FAC and organelle-specific trackers: MitoTracker (100 nM), LysoTracker (100 nM), or ER-Tracker (100 nM) for different times. Scale bars 20 μm.

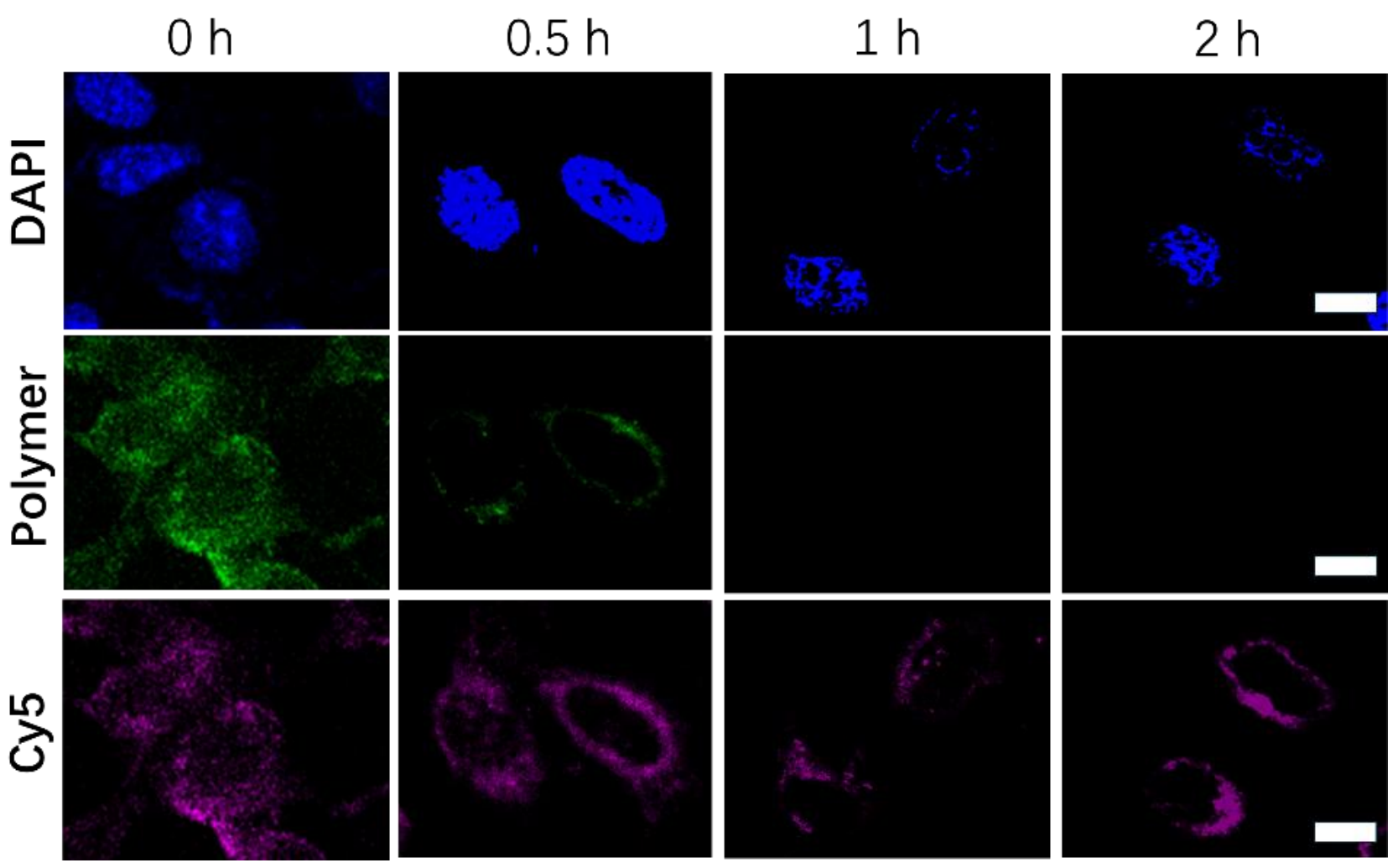

Figure S25. CLSM images of MCF-7 cells pretreated with Cy5-PUA (0.5 mg/mL) after 0.5, 1, and 2 h of incubation with FAC. Scale bars: 20 μm.

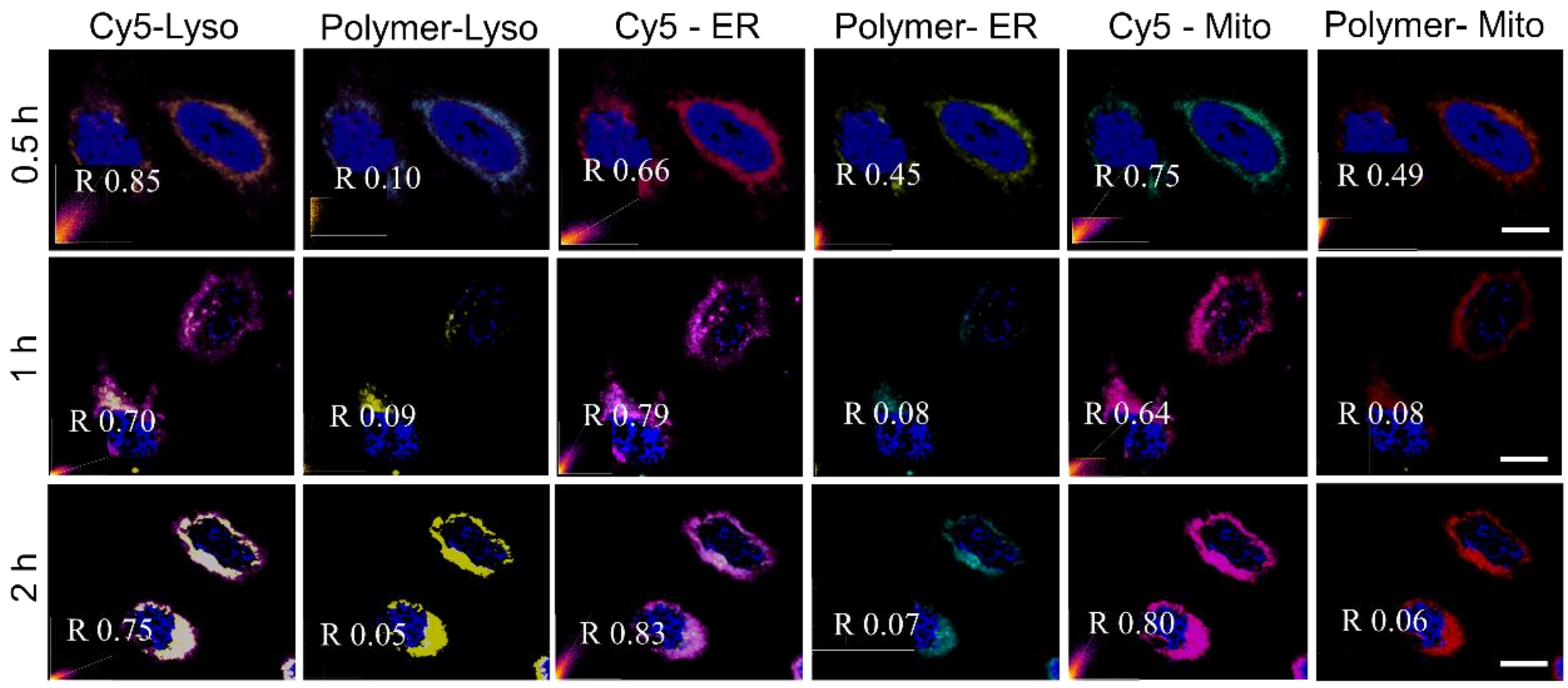

Figure S26. CLSM images of MCF-7 cells pretreated with Cy5-PUA (0.5 mg/mL) incubated with FAC and organelle-specific trackers: MitoTracker (100 nM), LysoTracker (100 nM), or ER-Tracker (100 nM) for different times. Scale bars 20 µm.

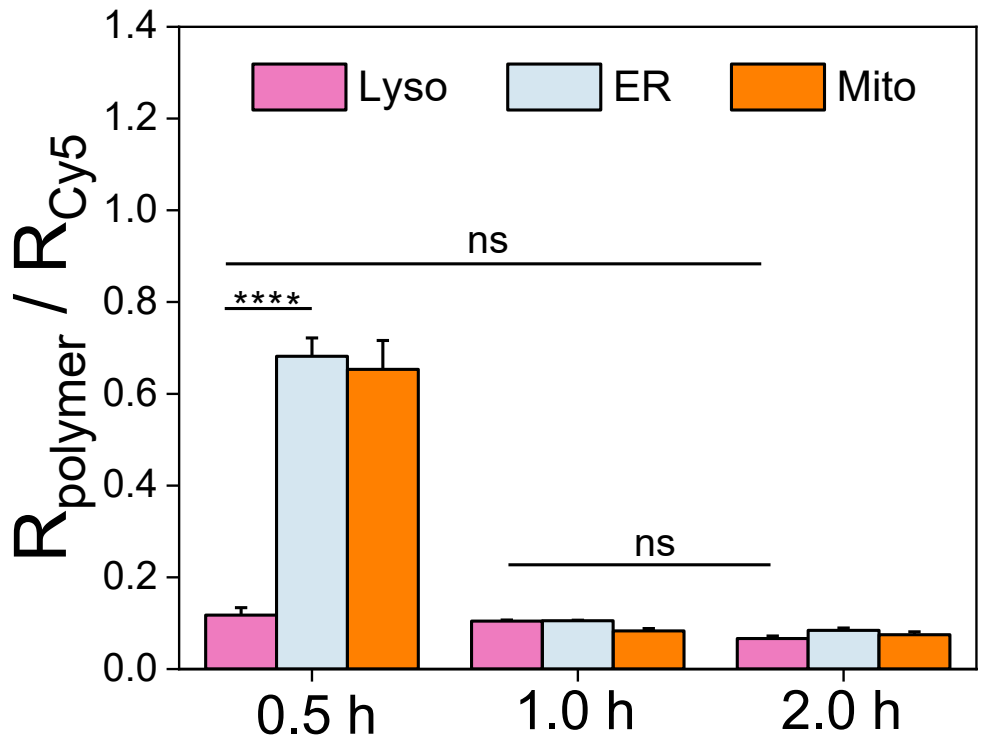

Figure S27. Temporal profiles of the mean fluorescence intensity ratio $R_{polymer}/R_{Cy5}$ of the corresponding subcellular imaging shown in Figure S26 by statistical analysis. Data are presented as mean ± standard deviation ($n = 3$ independent samples). Statistical significance was calculated by one-way ANOVA with Tukey's multiple comparisons test, ns : no significant; *: $p < 0.05$; **: $p < 0.01$; ***: $p < 0.001$; ****: $p < 0.0001$.

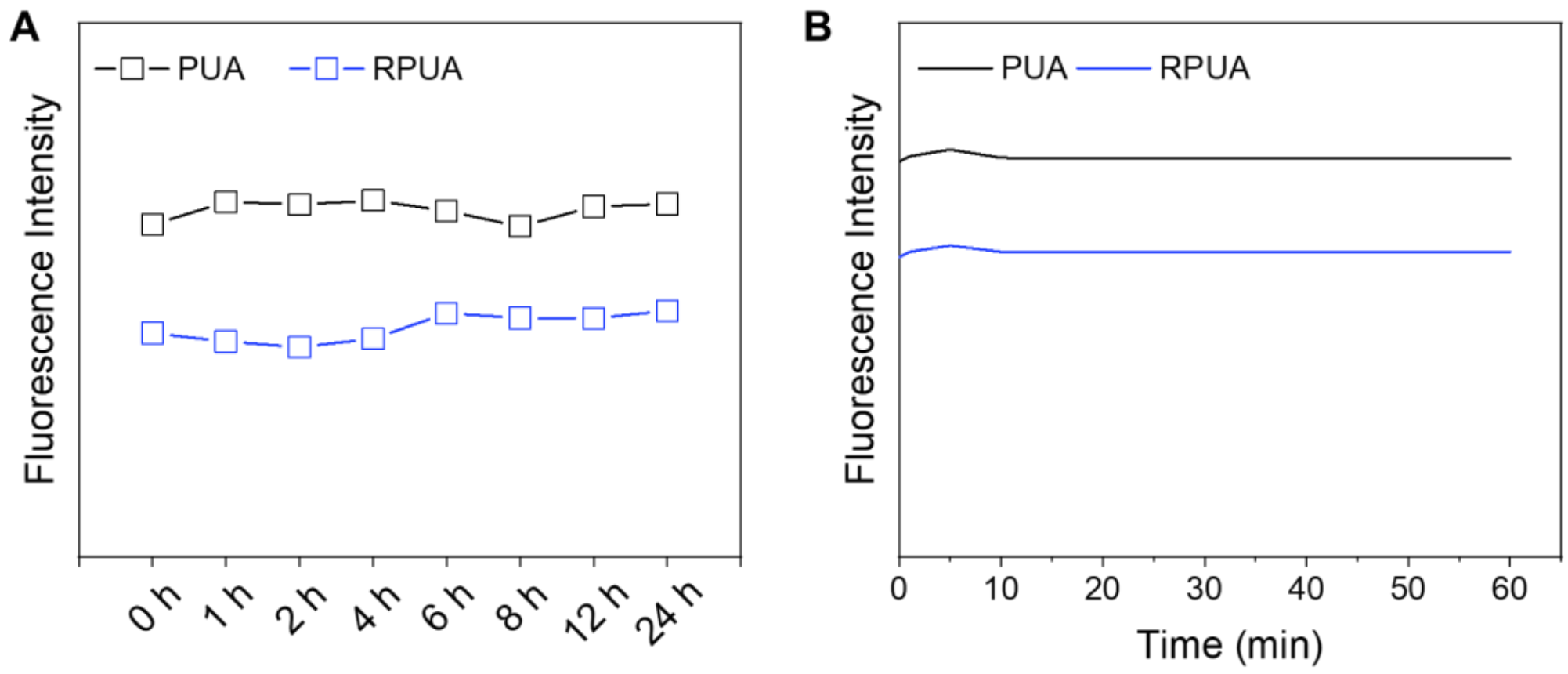

Figure S28. (**A**) Time evolution of the PL of polymer photoexcited under LED (20 W) irradiation. (**B**) Photobleaching fluorescent intensity of polymer under continuous irradiation.

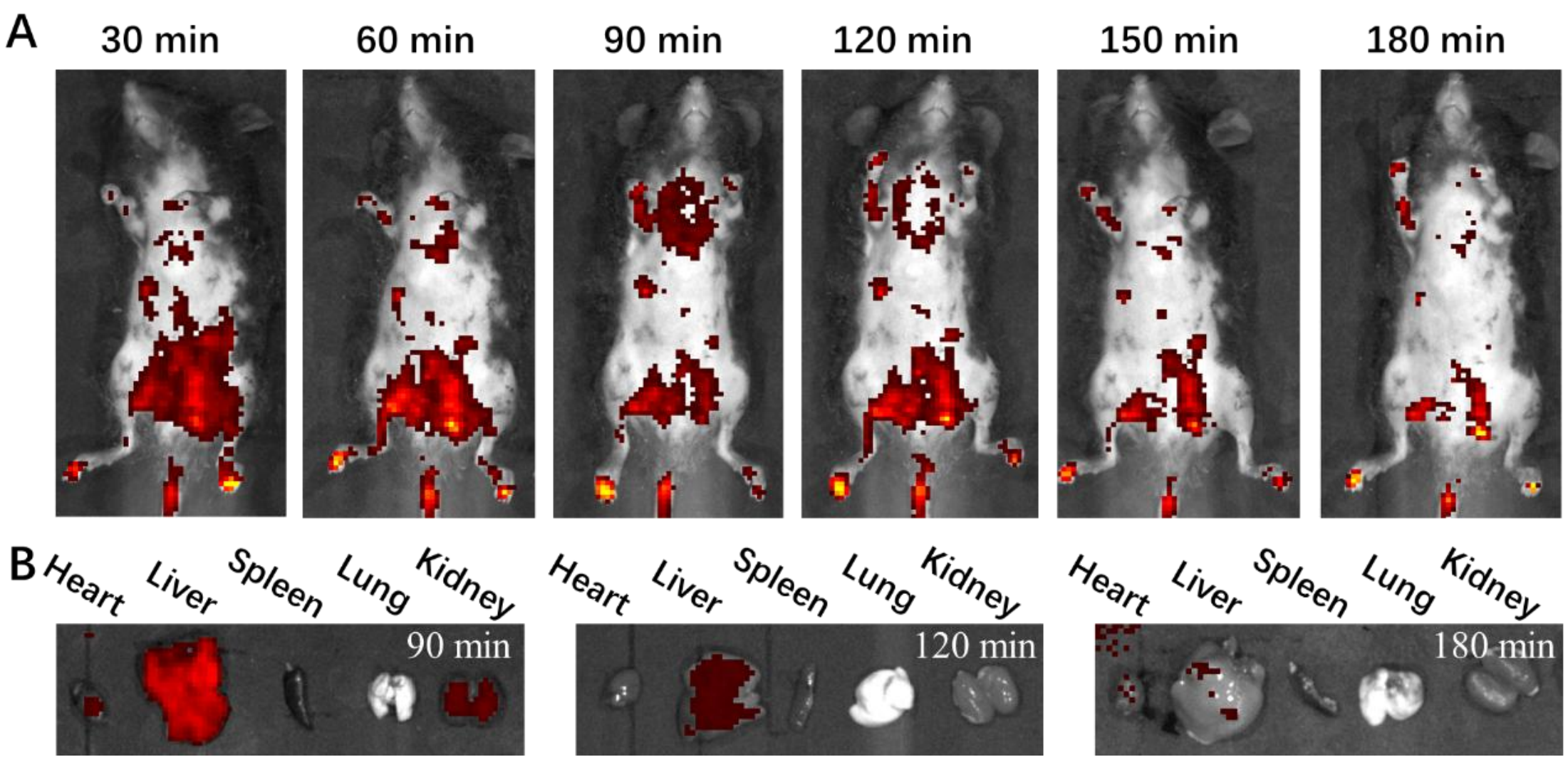

Figure S29. (**A**) In vivo fluorescence images at different time points after intraperitoneal injection of FAC following pretreatment with PUA, λ_{ex} = 480 nm, λ_{em} = 520 nm. (**B**) Ex vivo fluorescence images of major organs from mice in different groups at the indicated time points, λ_{ex} = 480 nm, λ_{em} = 520 nm.

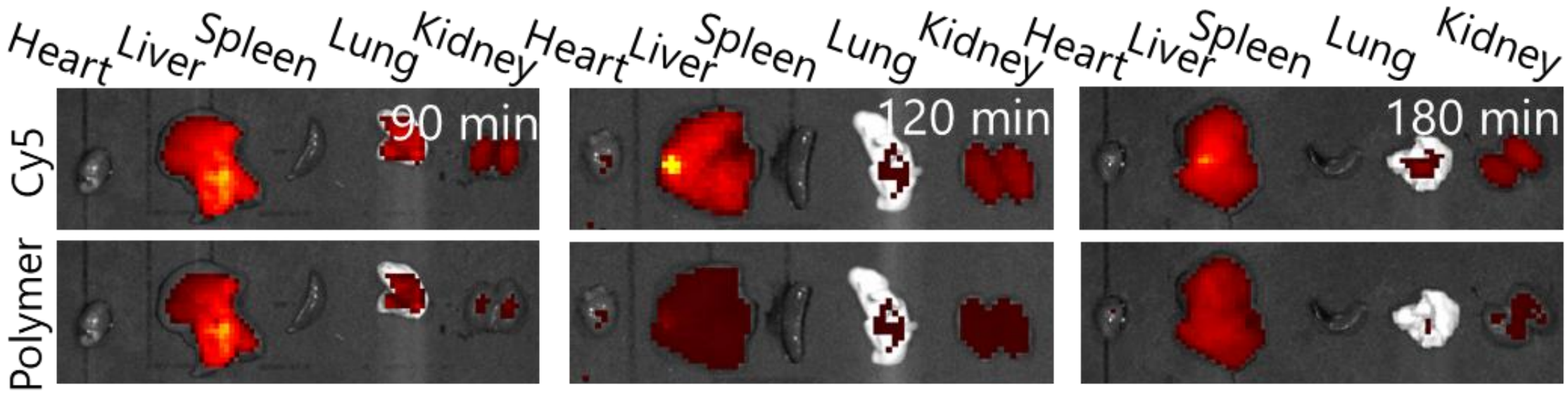

Figure S30. Ex vivo fluorescence images of major organs from mice in the RPUA group at the indicated time points, λ_{ex}= 480 nm, λ_{em} = 520 nm.

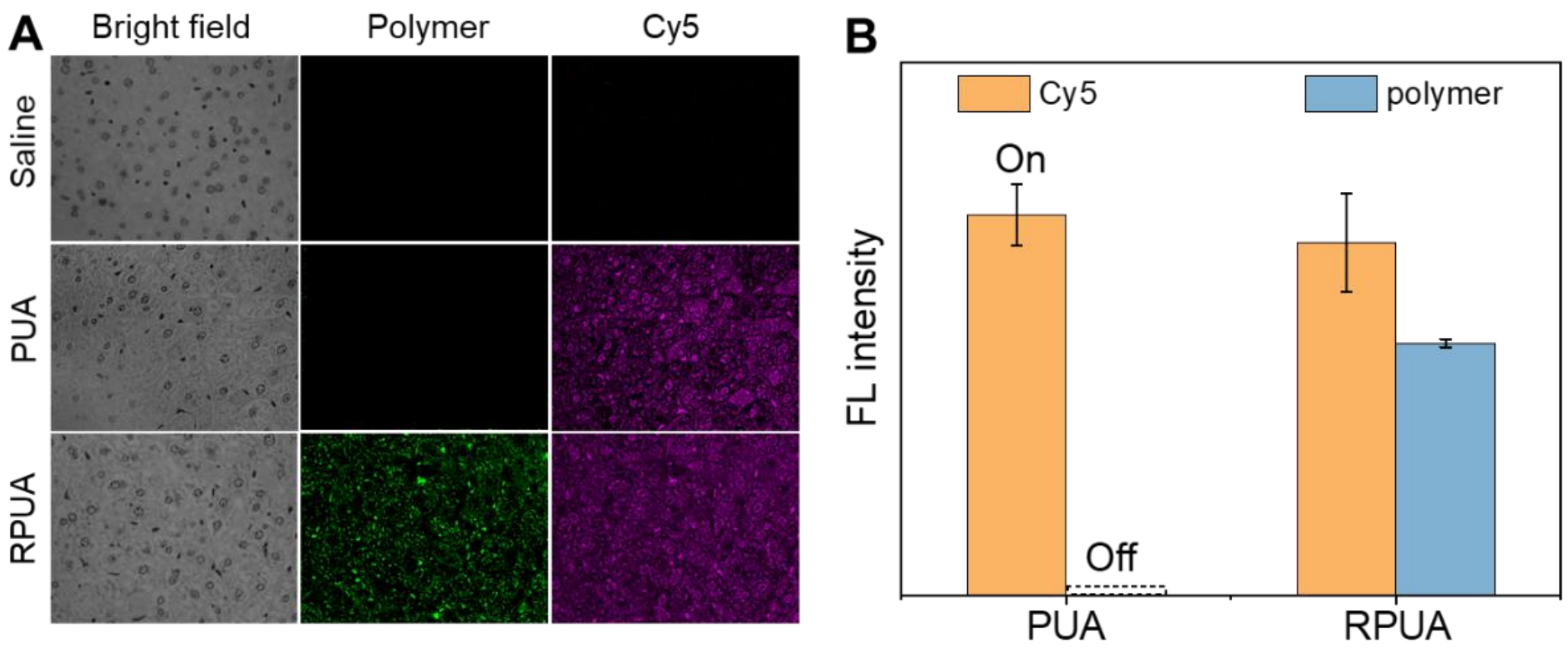

Figure S31. CLSM images of sectioned liver tissue (**A**) harvested at 180 min post-injection of FAC following pretreatment with PUA and RPUA. Saline, λ_{ex} = 480 nm, λ_{em} = 520 nm; PUA, λ_{ex} = 520 nm, λ_{em} = 790 nm; RPUA, λ_{ex}= 480 nm, λ_{em} = 520 nm. (**B**) Quantitative signal values of the corresponding liver tissue, represented as the mean ± SD.

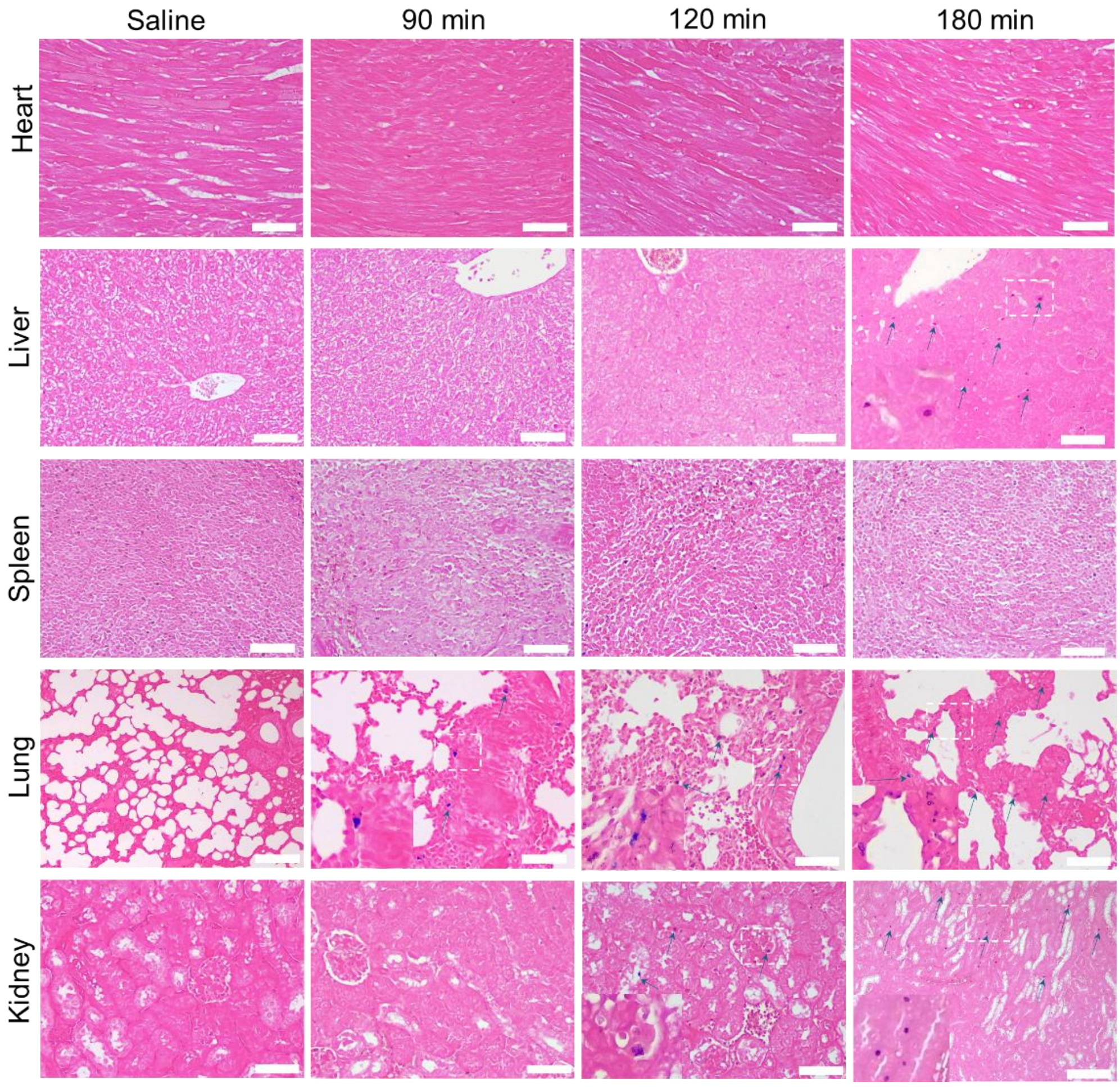

Figure S32. Representative images of major organ sections stained with Perls' Prussian blue from different treatment groups at indicated time points. Scale bar: 50 μm.

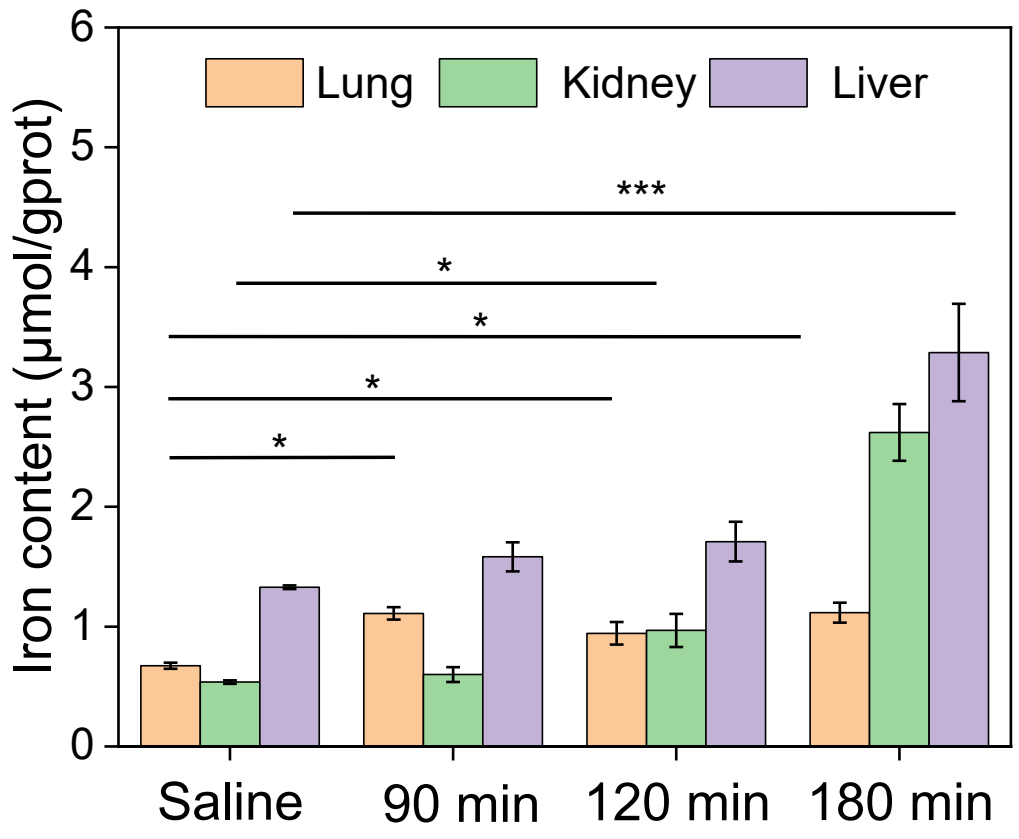

Figure S33. Quantification of iron content in mouse lungs, kidneys, and livers from different groups at the indicated time points, as determined by an iron colorimetric assay kit. Data are presented as mean ± standard deviation ($n = 5$ independent samples). Statistical significance was calculated by one-way ANOVA with Tukey's multiple comparisons test or a two-tailed paired Student's t-test. ns: no significance; *: $p < 0.05$; **: $p < 0.01$; ***: $p < 0.001$; ****: $p < 0.0001$.

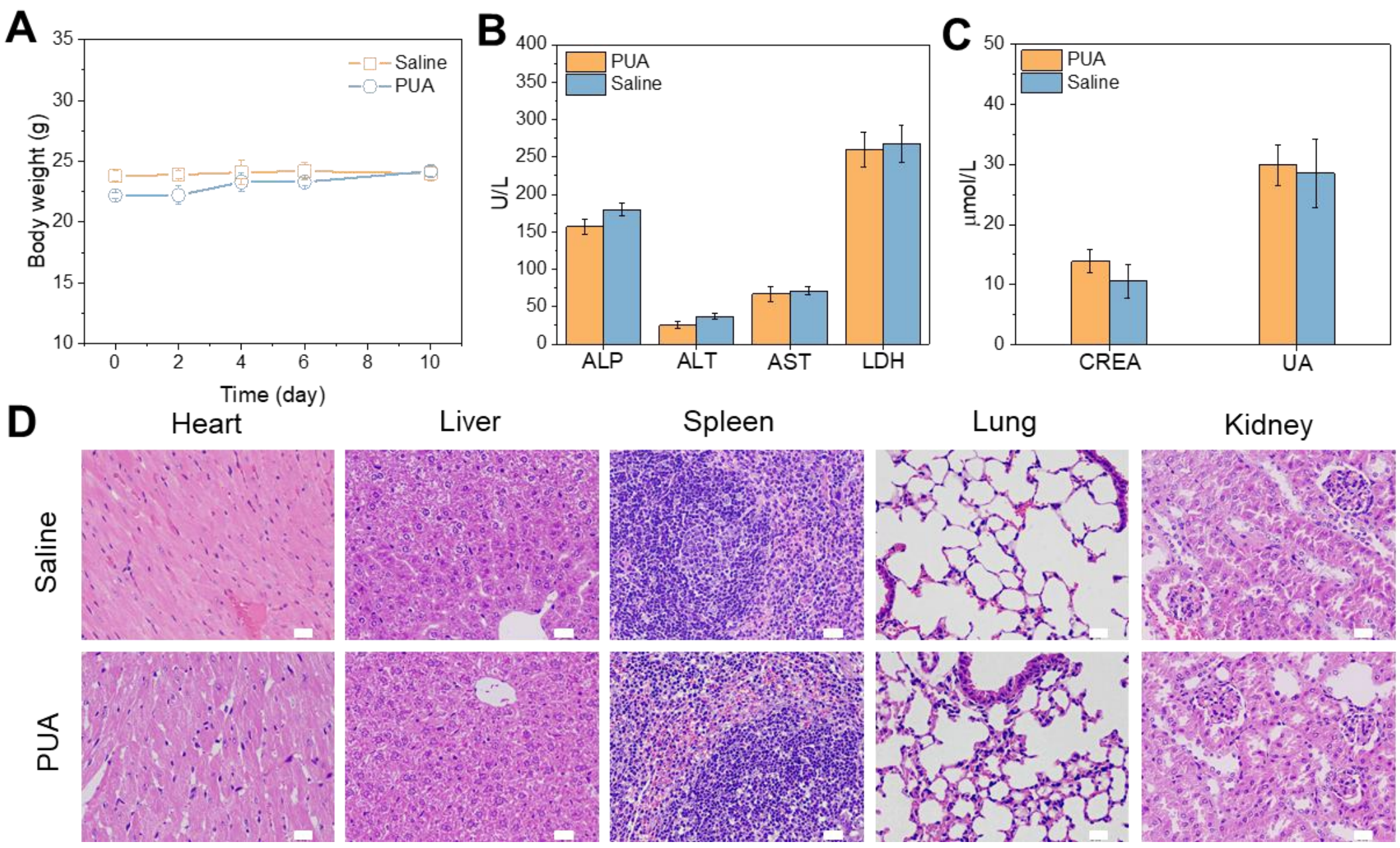

Figure S34. (**A**) Weight changes of healthy mice over 10 d post-injection of saline or PUA at a dose of 20 mg/kg. (**B**) Biochemistry analysis of renal-function-related indexes: creatinine (CREA) and uric acid (UA). (**C**) Biochemistry analysis of liver-function-related indexes: alkaline phosphatase (ALP), alanine aminotransferase (ALT), aspartate aminotransferase (AST), and lactate dehydrogenase (LDH). (**D**) Histological analysis of H&E-stained sections of major organs from mice at 10 d post-injection. Tissue paraffin sections were 4 μm thick and observed under light microscopy. Scale bars: 20 μm. Data are presented as mean ± SD.

Table S1 The molecular weight of the PUA and RPUA measured by ^{1}H NMR.

Sample	Integration values (A) of position (chemical shift)			C^b	L^b	M_n^b	M_i^c
	20 (1.87 ppm)[a]	15 (3.65 ppm)	16 (4.07 ppm)				
PUA	3	110.21	70.59	36.73	35.29	17503.84	15527.60
RPUA	3	113.13	69.98	37.71	35.00	17360.00	15400.00

[a]Integral value of area IEM (-CH_3, chemical shifts at 1.87 ppm) was calculated for a reference. [b]The number of Cys·OMe residues (C), Lys·OEt residues (L) and molecular weights (M_n) of PUAE and RPUAE were determined by ^{1}H NMR data. [c]The molecular weights (M_i) of PUA and RPUA was obtained by ^{1}H NMR.

Table S2. C=O···H-N and C=O···C=O distance of polymers in Figure. 1H.

Conformations	*d* (C=O···H-N)				*d* (C=O···C=O)		
Random coil	d_1	d_2	d_3	d_4	d_5	d_6	d_7
	3.51	4.86	7.95	7.95	6.88	6.84	6.89
Sheet	d_1	d_2	d_3	d_4	d_5	d_6	d_7
	2.73	3.48	2.84	3.50	5.71	5.16	3.78
Helix	d_1	d_2	d_3	d_4	d_5	d_6	d_7
	10.64	11.28	7.41	5.65	6.38	7.48	6.36
	d_8	d_9	d_{10}	d_{11}			
	2.62	3.12	3.20	3.43			

The d_1~ d_4 represent distance between NH in the urea linkage and the C=O of the carboxyl group. The d_5~ d_7 represent distance between C=O in the urea group and the C=O of the carboxyl group. The d_8~ d_{11} represent distance between NH and C=O in the urea group.
